# Piscine orthoreovirus demonstrates high infectivity but low virulence in Atlantic salmon of Pacific Canada

**DOI:** 10.1038/s41598-019-40025-7

**Published:** 2019-03-13

**Authors:** Mark P. Polinski, Gary D. Marty, Heindrich N. Snyman, Kyle A. Garver

**Affiliations:** 1 Pacific Biological Station, Fisheries and Oceans Canada, Nanaimo, V9T 6N7 Canada; 2 Animal Health Centre, Ministry of Agriculture, Abbotsford, V3G 2M3 Canada

## Abstract

Piscine orthoreovirus (PRV) is ubiquitous in farmed Atlantic salmon and sometimes associated with disease – most notably, Heart and Skeletal Muscle Inflammation (HSMI). However, PRV is also widespread in non-diseased fish, particularly in Pacific Canada, where few cases of severe heart inflammation have been documented. To better understand the mechanisms behind PRV-associated disease, this study investigated the infection dynamics of PRV from Pacific Canada and the potential for experimental passage of putatively associated heart inflammation in Pacific-adapted Mowi-McConnell Atlantic salmon. Regardless of the PRV source (fish with or without HSMI-like heart inflammation), infections led to high-load viremia that induced only minor focal heart inflammation without significant transcriptional induction of inflammatory cytokines. Repeated screening of PRV dsRNA/ssRNA along with histopathology and gene expression analysis of host blood and heart tissues identified three distinct phases of infection: (1) early systemic dissemination and replication without host recognition; (2) peak replication, erythrocyte inclusion body formation and load-dependent host recognition; (3) long-term, high-load viral persistence with limited replication or host recognition sometimes accompanied by minor heart inflammation. These findings contrast previous challenge trials with PRV from Norway that induced severe heart inflammation and indicate that strain and/or host specific factors are necessary to initiate PRV-associated disease.

## Introduction

Piscine orthoreovirus (PRV) infections of ocean-farmed salmon are widespread, and most farmed populations probably become infected at some point during a production cycle. These infections usually occur in the absence of overt disease; however, in some farming instances PRV has been associated with the development of serious disease syndromes. Specifically, a PRV subtype from Norway has been demonstrated to be the etiological agent of a disease known as Heart and Skeletal Muscle Inflammation (HSMI) of farmed Atlantic salmon (*Salmo salar*)^1^. This disease represents one of the most significant infectious diseases currently affecting Norwegian Atlantic salmon production^2^. Alternative PRV subtypes have also been identified in association with erythrocytic inclusion body syndrome (EIBS) in farmed Japanese Coho salmon (*Oncorhynchus* *kisutchi*)^3^ as well as with an HSMI-like disease in farmed Norwegian rainbow trout (*Oncorhynchus mykiss*)^4^; both conditions have been associated with high morbidity/mortality in some situations.

In contrast, PRV is also prevalent in many asymptomatic farmed salmon, particularly along the Pacific coast of North America, which often have high viral loads without associated lesions^5^. Indeed, despite the presence of PRV in farmed Atlantic salmon of British Columbia, Canada, for more than 30 years with presumed high prevalence during much of that time^5^, heart and/or skeletal muscle inflammation has not had a significant impact to farm production^6^. Two studies have reported HSMI-like disease in Pacific Canada^7,8^ (originally reported as HSMI but we say ‘HSMI-like’ as PRV etiology is uncertain). However, the condition is generally rare and not all fish diagnosed with these HSMI-like lesions were infected with PRV^7^ – a circumstance that has also been described previously^9^. Further, clinical presentation of disease as originally used in the case definition of HSMI as it occurs in Norway^10,11^ has never been reported in Pacific Canada.

A juxtaposition in virulence following PRV infection has also been highlighted in laboratory exposure trials. In Norway, exposure of Atlantic salmon to PRV by intra-peritoneal injection, cohabitation, and anal intubation have all resulted in moderate to severe heart lesions between 4–8 weeks post challenge^1,12^. In Canada, comparable PRV exposure trials using both intra-peritoneal injection and cohabitation have resulted in systemic PRV loads that were similar (if not greater) than those achieved in Norway, but no heart or skeletal muscle inflammation occurred during a similar time frame in either Atlantic or Sockeye (*Oncorhynchus nerka*) salmon^13^. Thus, it is currently unclear whether the HSMI-like lesions observed in Atlantic salmon of Pacific Canada are indeed HSMI; i. e., initiated primarily as a result of PRV.

One notable difference between Norwegian and Pacific Canada PRV exposure trials concerns the disease state of the donor fish from which PRV was sourced. Where Norwegian-based experiments have used PRV collected from Atlantic salmon with HSMI, Pacific Canada studies have used virus from non-diseased fish owing to the lack of regional availability for HSMI-diseased specimens. Thus, the previous inability of PRV from Pacific Canada to cause HSMI or any other disease symptoms in Atlantic salmon following challenge might be a result of genetic differences that have rendered at least some PRV variants of Pacific Canada less virulent. The fact that PRV sequenced from Pacific Canada to date appears phylogenetically distinct relative to PRV from Norway, which encompasses multiple amino-acid substations on various genomic segments, provides evidence in support of such a hypothesis^14^. However, the HSMI-like disease reported in PRV-infected Atlantic salmon from Pacific Canada supports an alternate hypothesis that at least one PRV variant from Pacific Canada may have caused severe heart inflammation in farmed Atlantic salmon of western North America^7,8^.

In this study, we compared PRV of Pacific Canada derived from two sources: (1) non-diseased fish and (2) fish with significant inflammation in the heart and skeletal muscle characteristic of HSMI. Our goal was to identify (i) if differences in virulence could be explained by genetic differences in the virus, and (ii) if, like in Norwegian studies, HSMI-like lesions as they have been recently diagnosed in Pacific Canada could be demonstrated as an infectious and transmissible disease within farmed Atlantic salmon. Further, we sought to improve upon the general understanding of PRV infection dynamics within farmed Atlantic salmon of Pacific Canada in the hope of identifying differences from Norway challenge trials that might help to explain the mechanisms for PRV virulence and pathogenesis.

## Results

### Preface concerning the diagnosis of HSMI

The original case definition of HSMI in Norway was founded on a set of clinical disease, gross pathology, and histological characteristics that could be differentially diagnosed from other common transmissible muscular disorders (e.g., cardiomyopathy syndrome and pancreas disease) using histopathology^10,11^. Currently, histolopathology is still used to diagnose clinical HSMI and also to differentially diagnose subclinical cases of HSMI, cardiomyopathy syndrome (CMS) and pancreas disease (PD) in farmed Atlantic salmon of Norway^15^. Although these diagnoses are made solely upon histological evaluations, it is generally accepted that the primary agent responsible for each disease is a unique virus: PRV is the primary agent responsible for HSMI^1^, piscine myocarditis virus (PMCV) is the primary agent responsible for CMS^16^, and salmon alpha virus (SAV) is the primary agent responsible for PD^17^. Indeed, although environmental and/or host contributing factors may explain the often exacerbated severity of HSMI in a field relative to laboratory setting, PRV appears to be the sole infectious agent associated with the unique set of histopathological criteria that defines HSMI in Norway^1,15,18^ and to our knowledge HSMI has never been used to classify a disease state in Norway where PRV has been confirmed to be absent.

Two recent studies from Pacific Canada have also used the term HSMI to classify subclinical heart disease of farmed Atlantic salmon based on histopathology in accordance with their own definitions that are similar to those previously reported in Norway – namely, moderate to severe heart inflammation sometimes accompanied by skeletal muscle inflammation^7,8^. Although the presumed commonality for the heart and skeletal muscle lesions in these Canadian studies relative to HSMI diagnosed in Norway is the causation by PRV, there is far less evidence in Canada to support that PRV is indeed the key component for initiating this disease state. Particularly given that these modified definitions based on heart pathology alone have occasionally been observed in the absence of PRV^5,7^. Consequently, if HSMI diagnosis is based solely on histopathological heart lesions which can occur in the absence of PRV, then PRV cannot be assumed to be *the* causative agent of the disease, but rather one of multiple stand-alone or synergistic putative factors.

For this study, we reserve the designation of HSMI in field environments to cases as defined by Wiik-Nielsen and colleagues^15^ – presence of cellular epicarditis; moderate-to-severe inflammation and necrosis especially in the ventricle (inflammation predominant); inflammation of the red skeletal muscle a supportive finding – for which PRV is the likely primary etiologic agent. We use the term HSMI-like for cases which fit the definition of HSMI as presented above but have questionable etiology, and use the term idiopathic cardiopathy in all other instances of heart associated pathology for which PRV may or may not be a contributing cause. In a laboratory setting where external factors are controlled, we adopt the modified diagnosis of HSMI previously used in assessing most experimental challenge trials in Norway; namely, histological visualization of moderate to severe heart inflammation that may or may not be accompanied by skeletal muscle inflammation but is clearly associated with a PRV infection, e.g., absent from the control population^1,19–22^.

### Case Report for HSMI-like disease in farmed Atlantic salmon of Pacific Canada

Idiopathic cardiopathy of a severity to cause morbidity or death (for which putative HSMI or HSMI-like lesions would be encompassed as a subset) is uncommon among British Columbia farmed Atlantic salmon and has historically been diagnosed in 1–3% of surveillance samples taken from moribund or recently deceased fish on farm sites since the early 1990s^23–26^. More recently, Fisheries and Oceans Canada Aquaculture Management Division conducts a regulatory Fish Health Auditing and Surveillance Program that from 2014–2017 sampled 2,960 moribund or recently deceased farmed Atlantic salmon for histopathology (https://open.canada.ca). These fish were collected during 407 audits of Atlantic salmon marine farms. Various types of idiopathic cardiopathy were diagnosed as a cause or marker of death in 62 (2.1%) of the sampled fish, and one of these fish (0.03% of 2,960) also had moderate skeletal muscle inflammation. Although some of the idiopathic cardiopathy observed in these samples (as well in samples from the early 1990s) might represent HSMI caused by PRV, a definitive diagnosis of HSMI is impossible to provide with any degree of certainty in these isolated occurrences.

However, on July 5^th^, 2016, microscopic lesions consistent with the recent diagnosis of HSMI in Pacific Canada^8^ were identified during a pre-transfer government audit of an Atlantic salmon sea-pen cohort in the Johnstone Strait off the eastern coast of Vancouver Island. Of the 40 fish sampled, 11 (28%) had inflammatory heart lesions within the atrium and ventricle that were moderate (10 fish) to severe (1 fish); two fish (5%) also had moderate inflammation within the red skeletal muscle. Between July 29^th^ and August 7^th^, 2016, 5 of 11 fish (45%) from the same farm were also diagnosed with moderate to severe heart inflammation characteristic of HSMI, although these samples did not have skeletal muscle inflammation. In a final sampling taken specifically for this study on August 19^th^, 2016, 3 of 20 fish (15%) had moderate to severe heart inflammation (but no moderate or severe skeletal muscle inflammation) and all 20 were qPCR positive for PRV L1 RNA with a mean load of 9.0e^8^ (±6.9e^8^ SEM) reverse transcribed copies per mL blood. The pathologists (HNS and GDM) who initially assessed these samples, although blinded to PRV status, reported that the lesions were similar to descriptions of HSMI as it is currently diagnosed in Norway, which was confirmed by a third pathologist (Renate Johansen; Pharmaq Analytiq, Bergen Norway) with long experience diagnosing HSMI in farmed Atlantic salmon in Norway. A summary for the histopathology scoring of heart and muscle tissues from all three pathologists was highly comparable (Supplement 1).

All fish on the affected site were progeny of North America-adapted Mowi-McConnell brood stock^27^ and had been reared in a single freshwater facility on Vancouver Island, Canada. Fish were presumed to be PRV-free upon seawater entry because virus was not detected by qPCR within a companion cohort from the same freshwater hatchery where these fish were sourced (n = 20). The first sampling and diagnosis of HSMI-like lesions was 2–3 months post seawater entry. At sea, and specific to the farm and period where the lesions were diagnosed, the site veterinarian reports that the population did not have clinical signs of disease. Total farm monthly mortality (all causes) was between 0.2–0.5%, which is below the industry average of ~0.83%, and feeding behavior was normal with mean growth rates in accordance with company-targeted projections.

### PRV from Atlantic salmon with and without HSMI-like lesions generate extensive and persistent infections in naïve recipients

Naïve juvenile Atlantic salmon originating from the same brood stock and rearing facility as the fish with HSMI-like lesions described above were exposed in parallel under controlled laboratory conditions to PRV from one of two sources: (1) three fish with HSMI-like heart lesions collected August 19^th^ described above (designated 16-011D), and (2) three non-diseased fish infected by experimental challenge with PRV originating from an alternate freshwater facility with at least a three year history of PRV presence without documentation of heart or muscle inflammation; this PRV had not generated HSMI in previous laboratory challenge trials^13,28^ (designated 16–005ND; see Methods section).

**Figure 1:**
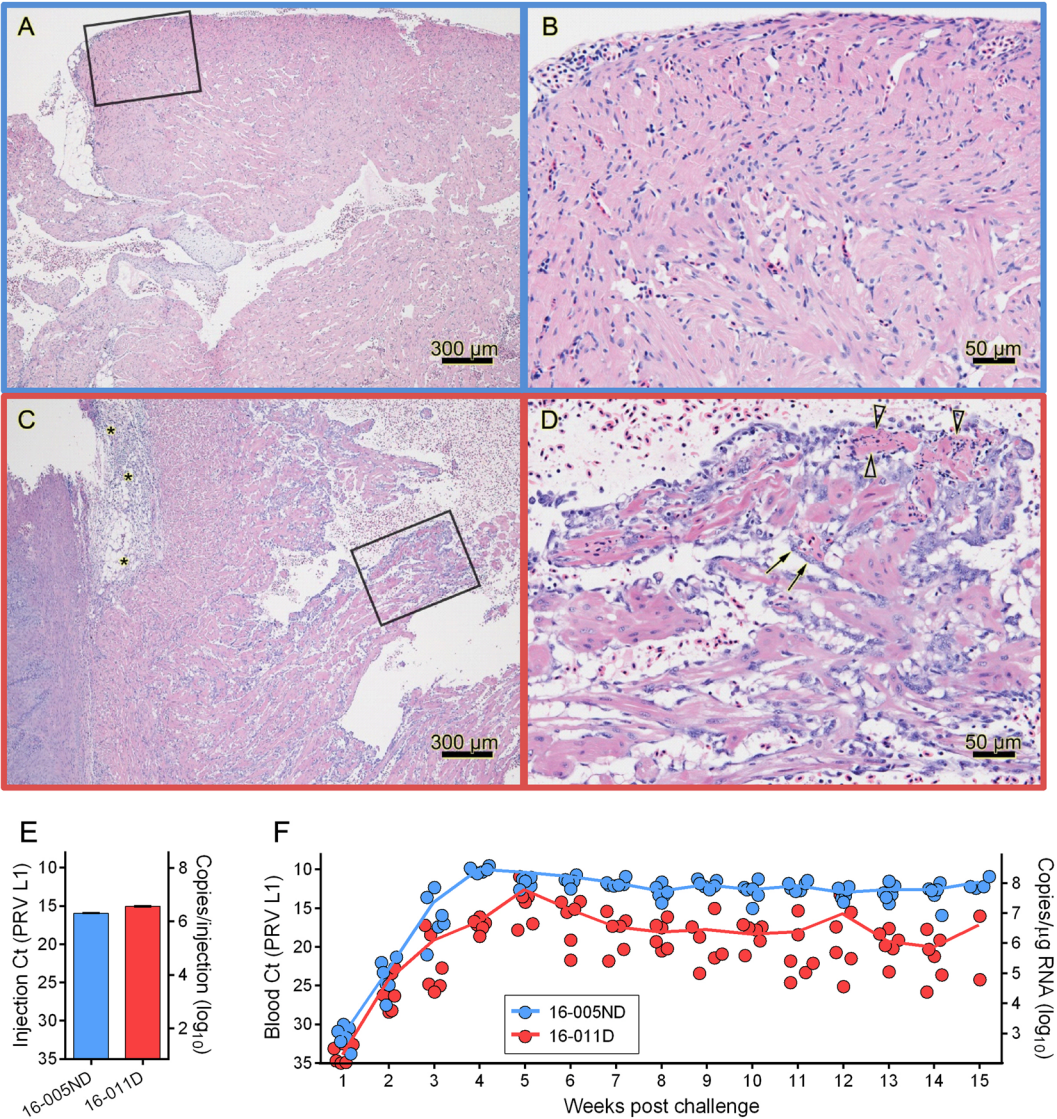
PRV from Atlantic salmon with and without HSMI-like lesions generate extensive and persistent infections in naïve recipients. (**A**,**B**) PRV was obtained from the blood of donor fish without heart lesions (16-005ND) or (**C**,**D**) with HSMI-like lesions (16-011D). Lesions in diseased fish included epicarditis (*), endocardial cell hypertrophy (arrows) and small foci of myocardial necrosis (open arrowheads). Black boxes within left images outline the area shown at higher magnification in right images. (**E**) The quantity of L1 transcripts of PRV 16-005ND and 16-011D inoculated into each naïve recipient was estimated by qPCR, and (**F**) the systemic blood load of PRV L1 transcripts was assessed every 7 days in recipient fish (n = 6 per treatment where available). The unique relative qPCR threshold cycle (Ct) associated with each sample (dots) as well as the mean estimated quantity of L1 copies per µg total RNA at each time point (lines) are shown.

Following intra-peritoneal (i.p.) injection of similar quantities of PRV, virus replication dynamics were slightly different for the two PRV inoculates. The total quantity of PRV L1 copies per injection was 2.0e^6^ for 16-005ND and 3.7e^6^ for 16-011D (determined by TaqMan qPCR; Fig. 1E) and both inoculates generated substantial systemic infections. However, PRV 16-005ND rapidly disseminated and replicated to peak transcriptional loads by four weeks post challenge (wpc) with 2.9e^8^ mean PRV L1 copies per µg RNA (Fig. 1F). This was estimated at >1.0e^11^ copies per mL blood based on the total RNA yield per 100 µL blood sample. After 4 wpc, systemic PRV transcripts decreased but remained substantial, with a mean load of 6.8e^7^ copies per µg RNA (>2.0e^10^ copies per mL blood) between 10-15 wpc. In contrast, infection with PRV 16-011D was slower to develop, with peak loads not occurring until 5 wpc and to a fivefold lesser degree (mean quantity of 6.0e^7^ PRV L1 copies per ug RNA) than observed for PRV 16-005ND. Further, although the PRV 16-011D challenge generated large quantities of virus with persistent high-load systemic infections – 3.2e^6^ mean copies per µg RNA, representing >1.3e^9^ copies per mL blood between 10–15 wpc – this persistent load was only about 5% of PRV 16-005ND.

### PRV sourced from cohorts with and without HSMI-like lesions have high sequence similarity

**Figure 2:**
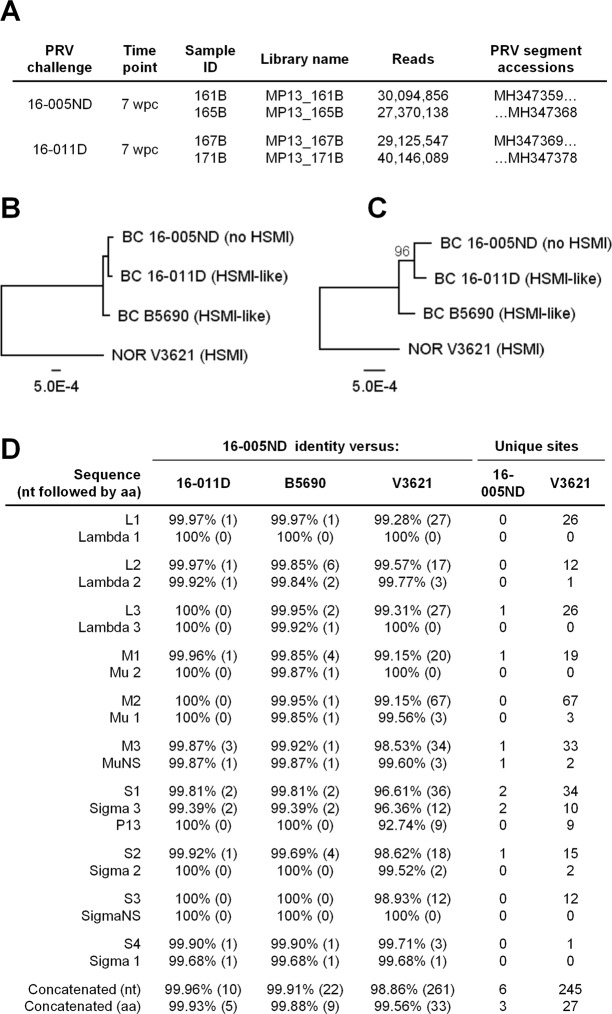
PRV sourced from cohorts with and without HSMI-like lesions have high sequence similarity. (**A)** RNA-seq read libraries (n = 4) were pooled specific to PRV source material (n = 2) and assembled *de novo* using Trinity. **(B)** Concatenated PRV segments are phylogenetically compared to two previously published genomes of PRV (Pacific Canada isolate B5690^8^ and Norway isolate V3621^1^). Bootstrap probabilities (percentages) are provided at branch nodes if less than 100%; scale bar indicates nucleotide substitutions per site. **(C)** Predicted amino acid sequences are compared in the same manner as nucleotide sequences. **(D)** The comparative identity between nucleotide (nt) and predicted amino acid (aa) sequences as well as the number of substitutions () per alignment are provided in relation to 16-005ND. Substitutions unique to either 16-005ND (no HSMI) or V3621 (only Norwegian isolate; HSMI) are also indicated.

RNA extracted and purified from the blood of both PRV 16-005ND and 16-011D challenged fish at 7 wpc (n = 2 per treatment) were subjected to next-generation RNA-sequencing (RNA-seq) to independently obtain the genomic sequences of PRV 16-005ND and 16-011D. Following rRNA depletion and Illumina sequencing, 27–40 million reads with Phred scores >33 were generated from the RNA of each fish and deposited in the NCBI Sequence Read Archive, study SRP145317. Individual SRA library accession numbers are provided in Fig. 2A. Libraries specific to either 16-005ND or 16-011D were pooled for *de novo* transcript assembly using Trinity^29^ which identified all 10 genomic segment of PRV for both isolates. The longest Trinity assembled PRV transcripts were deposited in Genbank; accession MH347359 – MH347378 (Fig. 2A). *De novo* assembled transcripts had 98.5% (16-005ND) and 98.9% (16-011D) concatenated coverage across all segments with respect to two previously published PRV genomes – one isolated from diseased Atlantic salmon in British Columbia during the first reported outbreak of HSMI in the region (isolate B5690)^8^, the other from HSMI diseased Atlantic salmon in Norway which was used to demonstrate laboratory passage of HSMI and the causal relationship of PRV in HSMI disease within Norway (NOR2012-V3621)^1^. This coverage incorporated 99.5% (16-005ND) and 100% (16-011D) of the protein coding sequence for these two published genomes where the only notable loss in coverage was the putative 128 bp (41 aa) missing 5′ end of the L1 segment for 16-005ND.

Genomic sequences of 16-005ND and 16-011D have 99.96% nucleotide identity with only 10 nucleotide substitutions over the approximate 23 kb concatenated genome. Both isolates are also highly similar (>99.9% nucleotide identity) to the B5690 isolate previously identified in BC, but are comparatively divergent (~98.9% nucleotide identity) to the Norwegian V3621 isolate (Fig. 2B–D). This pattern of divergence is similarly reflected in predicted protein translation. Specifically, V3621 has 245 unique nucleotide substitutions that results in 27 predicted amino acid changes relative to the three Canadian isolates considered in this study, with the highest proportion of translational variation being associated with the bicistronic S1 segment (Fig. 2D). However, only six nucleotide substitutions (resulting in 3 predicted amino acid substitutions) are unique to Pacific Canada PRV sourced from non-diseased Atlantic salmon (16-005ND) compared to isolates from HSMI diseased fish in Norway and fish with HSMI-like lesions in Pacific Canada. All three of these putative amino acid substitutions (one from MuNS, two from Sigma 3) are predicted to alter protein secondary structure as identified by the EMBOSS 6.5.7 garnier plugin within Geneious 9.1.7 software. The MuNS substitution is predicted to eliminate a coil at position 386 thereby extending an alpha helix. The substitution at position 180 of Sigma 3 results in the same alteration, whereas the substitution at position 230 of Sigma 3 results in the predicted shortening of a beta-strand and lengthening the subsequent alpha helix. These structural changes are predicted with approximately 65% accuracy and the consequences (if any) for these changes in secondary structure or putative protein function are unknown.

### Systemic PRV load is associated with erythrocytes and not detectable in plasma

**Figure 3:**
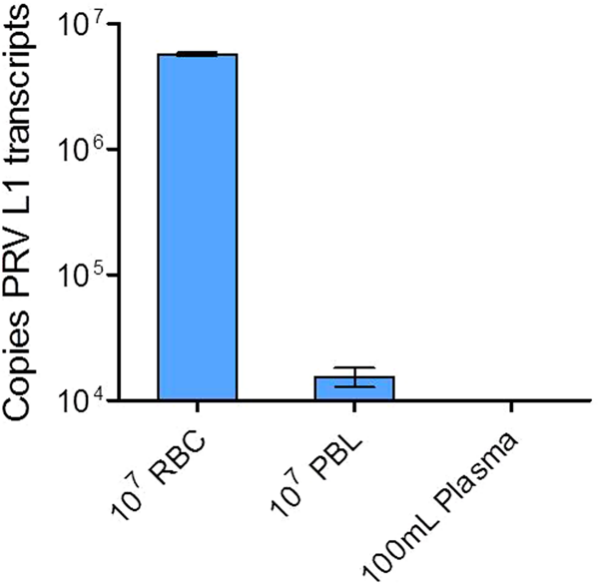
Systemic blood PRV load is associated with erythrocytes and not detectable in plasma. Copy number of PRV L1 transcripts at 10 weeks post PRV 16-005ND challenge (n = 3) was high within the erythrocyte red blood cell (RBC) fraction, low within the peripheral blood leukocyte (PBL) fraction, and not detectable in plasma.

At 10 wpc, a portion of blood collected from three 16-005ND PRV infected fish was separated into plasma, leukocyte, and erythrocyte fractions. By qPCR screening, virtually all PRV L1 transcripts were associated with the erythrocyte fraction during this persistent phase of infection (Fig. 3). Further, PRV L1 RNA was not detected in (i) plasma (limit of detection of approximately 30 copies per 100 µL) at the 10 wpc time point or (ii) from the plasma of any PRV-challenged fish (both 16-005ND and 16-011D) collected across all sampling time points.

### The relative proportion of PRV genomic and messenger RNA in erythrocytes changes over the time course of infection

**Figure 4:**
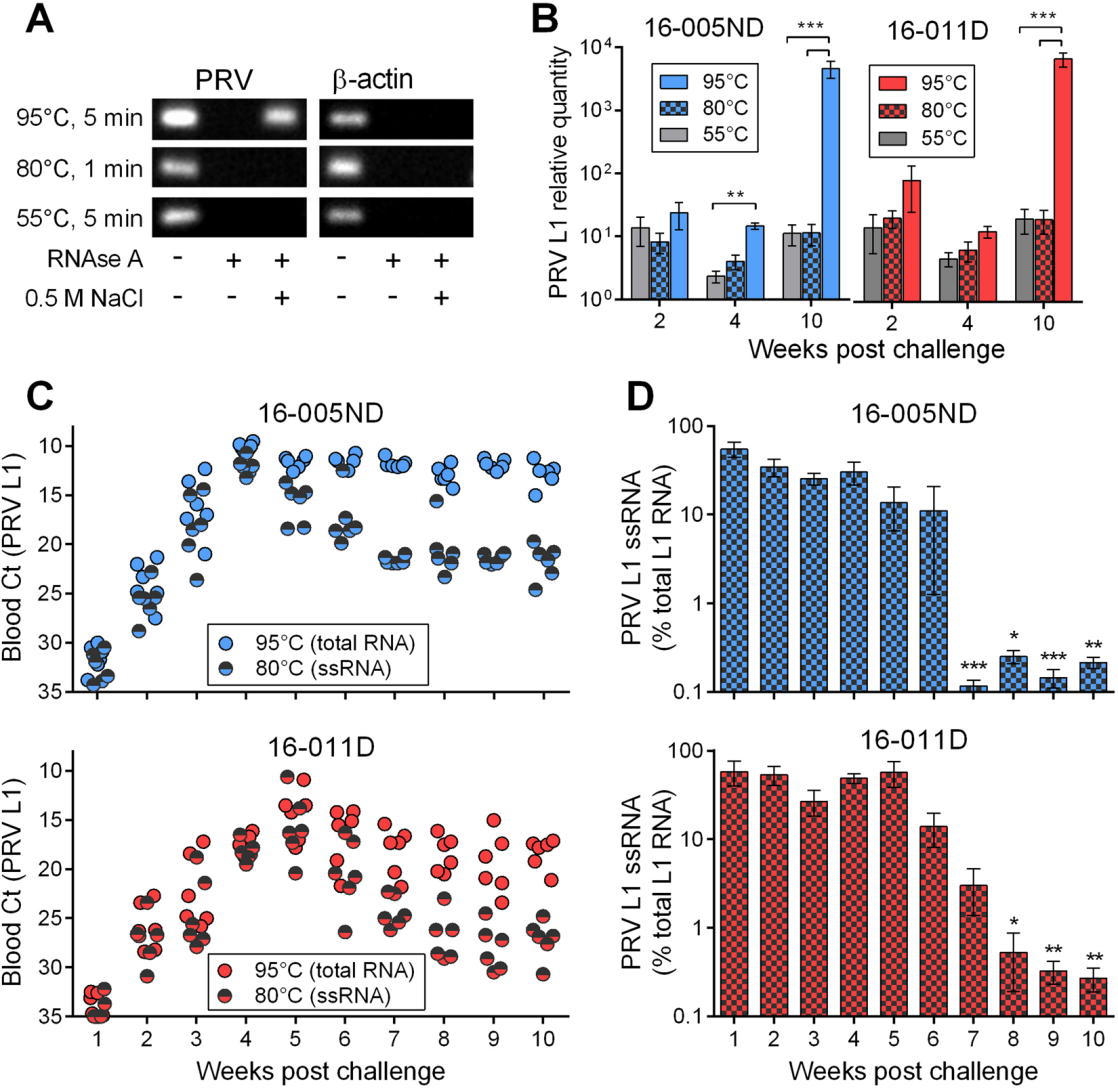
The relative proportion of PRV genomic and messenger RNA in blood changes over the time course of infection. (**A)** RNA from PRV 16-005ND infected fish at 10 wpc was used to validate differential detection of PRV L1 ssRNA (mRNA) and dsRNA (gRNA) transcripts by qPCR. Total RNA exposed to either no enzyme, RNAse A, or RNAse A in 0.5 M sodium chloride (which selectively protects dsRNA but not ssRNA from RNAse A degradation^60^) was heated at 55 °C for 5 min, 80 °C for 1 min, or 95 °C for 5 min prior to reverse transcription and 30-cycles of qPCR. PRV dsRNA template was only present following 95 °C denaturation as seen in cropped gel images relative to host β-actin ssRNA (for Ct values see Supplement 1). **(B)** The relative quantities (scaled to the minimum value per time point) of PRV L1 at 2, 4, and 10 wpc for both PRV 16-005ND and 16-011D are compared between pre-amplification denaturation temperatures. The proportion of dsRNA to ssRNA significantly increased at 10 wpc in both PRV challenged groups (*p < 0.05; **p < 0.01, ***p < 0.001). **(C)** The shift toward higher proportions of PRV dsRNA in blood began around 5-6 wpc; **(D)** where the amount of ssRNA became significantly reduced after 7-8 wpc relative to 1wpc proportional quantities.

Reoviruses have a dsRNA genome which is asynchronously transcribed. Positive-strand RNA is synthesized first which acts as mRNA template for viral protein translation and template for subsequent minus-strand synthesis of genomic material (gRNA)^30^. To better understand the timing and degree of PRV protein production versus gRNA synthesis, we developed a new method to differentially quantify PRV single-stranded (mRNA) and double-stranded (gRNA) RNA using qPCR. Because recombinant reverse transcriptases such as MultiScribe™ selectively target ssRNAs during cDNA synthesis, we hypothesized that PRV dsRNA segments would not be detected by qPCR unless they were denatured into single-strands prior to reverse transcription of cDNA and that this would require temperatures above 90 °C based on sequence-specific melting point estimations. By this reasoning, exposure to low temperatures (i.e., below 90 °C) for the removal of secondary structure prior to reverse transcription would result in only single-stranded PRV (mRNA) detection by qPCR. Experimental testing confirmed this hypothesis to be correct, as the dsRNA genomic component was only measureable in those samples denatured at 95 °C and not following exposure to either 55 or 80 °C (Fig. 4A). Capitalizing on the ability to differentiate mRNA vs gRNA via differential temperature screening regimes, we analyzed early (2 wpc), peak (4 wpc), and late (10 wpc) stages of PRV infection to compare relative transcription of mRNA and gRNA. Utilizing two temperatures (55° and 80 °C) below the threshold needed to denature dsRNA, the relative proportional quantity of PRV L1 ssRNA transcripts was not significantly different at either temperature. Yet, when measuring both mRNA and gRNA in the denatured samples (95 °C), the fraction of the gRNA differed significantly dependent upon sample time point. At 2 and 4 wpc the ssRNA component constituted approximately 50 (±40)% of the total RNA while at 10 wpc the ssRNA component was significantly reduced, only representing 0.1-0.7% of the total PRV RNA load (Fig. 4B). Subsequent targeting of total and ssRNA PRV L1 transcripts in all blood samples collected between 1 and 10 wpc confirmed that during early and peak replication, single-stranded mRNA typically represented 10–90% of the total PRV transcriptional load; but, after approximately 5–6 wpc, the quantity of single-stranded mRNA quickly became proportionally less. By 7–8 wpc, PRV ssRNA represented only 0.1–1.0% of the total systemic transcriptional load which was significantly less than during early (1wpc) infection (Fig. 4C,D). This pattern of expression was strikingly similar following challenge with either PRV 16-005ND or 16-011D.

### Expression of PRV segments is temporally similar but with slight proportional variation

**Figure 5:**
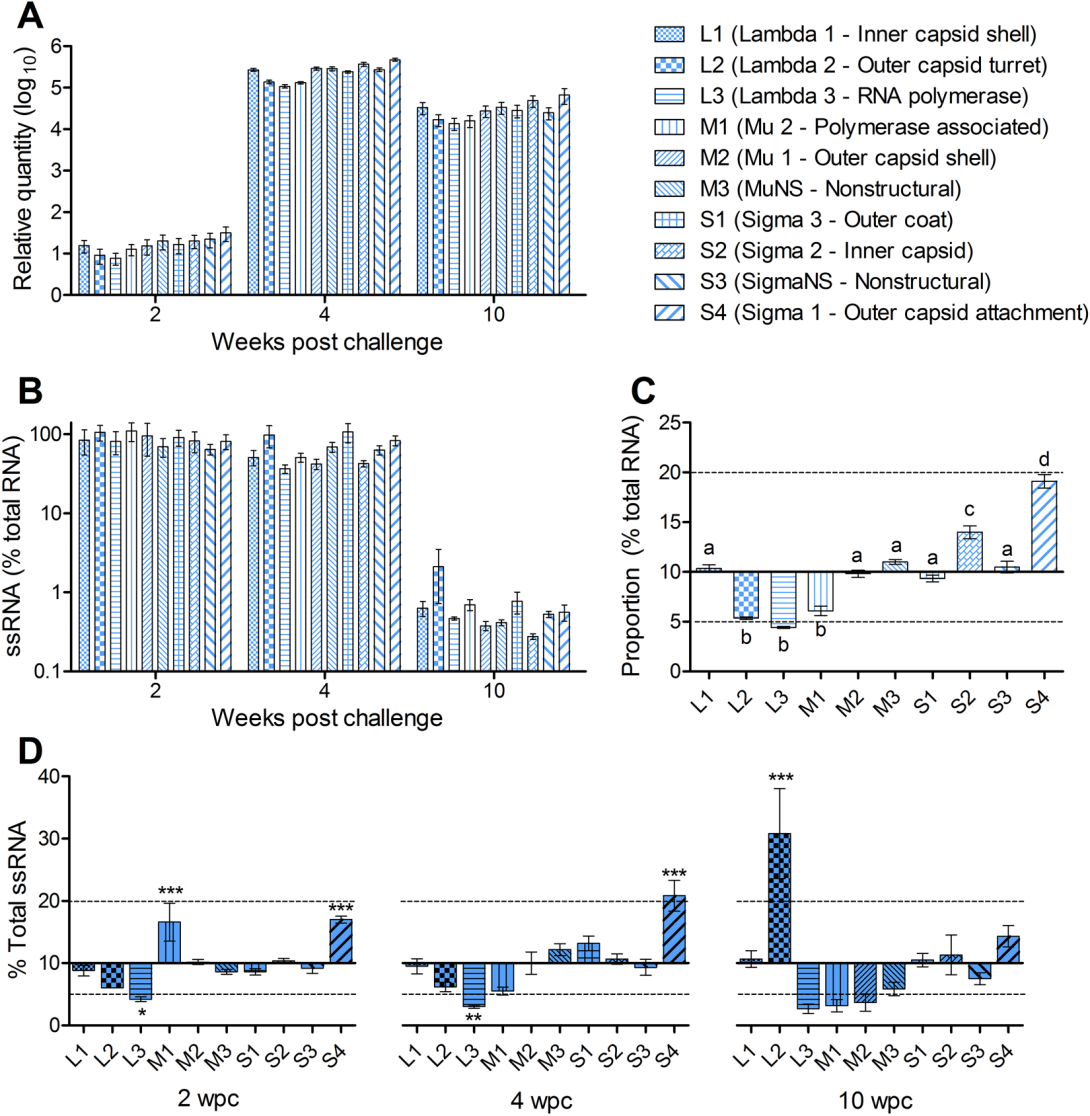
Expression of PRV segments in host blood is temporally similar but with slight proportional variation. (**A)** The relative quantity (scaled to the minimum value) of each PRV RNA segment in the blood of 16-005ND challenged fish were statistically similar at 2, 4 or 10 wpc. **(B)** The single-stranded mRNA contribution to total PRV load was also similar between segments at each time point. However, **(C)** the cumulative proportional contribution of L2, L3, and M1 was significantly less, whereas S2 and S4 was significantly more, relative to all other segments independent of time (letters indicate significant groupings at p < 0.05). **(D)** Total proportional contributions of total single-stranded mRNA expression also varied between segments, but was not consistent over time points (*p < 0.05; **p < 0.01; ***p < 0.001). Dotted lines at 5 and 20% provide reference of a twofold deviation away from complete proportional equality (10%).

To compare and contrast the relative load for each of the 10 PRV genomic segments following experimental infection, SYBR™ green qPCR assays were designed against each of the 10 genomic segments of PRV and used to assess relative quantities of each segment in the blood of PRV 16-005ND challenged fish at early (2 wpc), peak (4 wpc) and persistent (10 wpc) phases of infection. This identified that relative quantities of all 10 segments of PRV 16-005ND were not significantly different at any of the three time points analyzed even though the total systemic PRV RNA load encompassed an approximate 10e^4^ fold change over this time (Fig. 5A); indicating the proportion of total PRV RNA represented by each segment was conserved during all three infection stages. However, the proportion of each segment contributing to the total PRV RNA independent of time was not equal; this variation was less than twofold but statistically significant (p < 0.05). Specifically, L2, L3 and M1 were proportionally underrepresented whereas S1 and particularly S4 were overrepresented relative to the other segments (Fig. 5C).

Selective ssRNA targeting techniques previously validated for L1 were also applied to each of the other 9 segments in this dataset. In a similar pattern to L1 transcription, the ssRNA component of all 10 PRV segments was relatively high (~10–90%) during early and peak infection but became reduced (nearly all < 1%) following the transition to late-stage persistent infections (Fig. 5B). Nevertheless, significant proportional variation in ssRNA quantity occurred for some segments over the course of infection. In comparison to segment L1 ssRNA, which maintained stable expression across all three time points (mean 9.7% ± 0.7 SEM of total ssRNA), segment L3 ssRNA was proportionally decreased whereas segment S4 was proportionally increased during early and peak infection. Segment M1 ssRNA was also proportionally increased during early infection, whereas segment L2 ssRNA only became proportionally increased during the late persistent phase (Fig. 5D). L1, M2, M3, S1, S2 and S3 maintained relatively stable proportional ssRNA expression across all three time points; and even though L2, L3, M1 and S4 had significant proportional variation compared to the other stable segments, their variation was mostly encompassed within a twofold change relative to complete segment equality (10% each).

### Persistent late-stage PRV infections remain highly infectious by i.p. injection but have reduced ability for infecting naïve cohabitants

**Figure 6:**
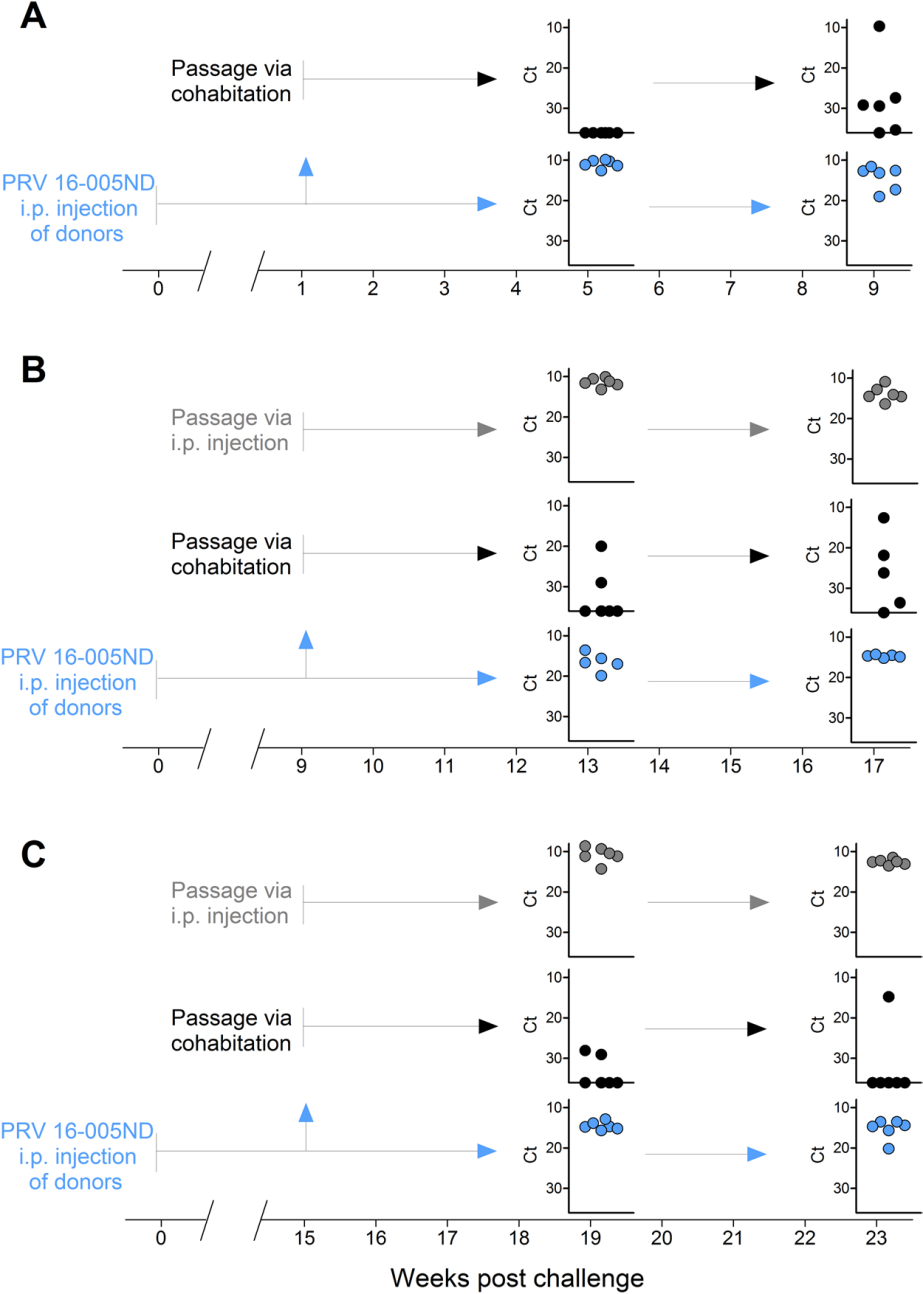
Persistent late-stage PRV blood infections remain highly infectious by i.p. injection but have reduced ability for infecting naïve cohabitants. Passage of PRV 16-005ND from i.p. injected donor fish into naïve recipients by either i.p. injection or cohabitation was attempted at (**A**) 1, (**B**) 9, and (**C**) 15 wpc. After a period of 4 or 8 weeks following each attempted passage, blood of both recipients and donor fish (n = 6 per group) was screened for total PRV L1 transcripts by qPCR. The unique relative qPCR threshold cycle (Ct) associated with each sample is presented. Samples plotted on x-axes indicate a lack of detection for PRV L1 transcripts in each instance (no Ct).

The limited quantities of PRV single stranded mRNA at 10 wpc indicated that viral replication had become substantially reduced. To determine the transmission potential of PRV during different stages of infection, we initiated a third challenge trial where three groups of naïve fish (n = 15 per group) were injected with PRV 16-005ND from which viral passage was attempted by either cohabitation (1:1 shedder to naïve fish ratio) or by i.p. injection (100 uL blood homogenate diluted 1:10 in saline) to naïve recipients introduced at either 1, 9, or 15 weeks post challenge (Fig. 6). Fish injected with PRV 16-005ND had infection dynamics similar to those observed in the first challenge. Viral loads were high at 5 wpc (6.2e^7^ mean copies per µg RNA), were slightly less at 9 wpc, and stabilized at reduced but still substantial loads at 13, 17, 19 and 23 wpc (4.9e^6^ mean copies per µg RNA). Passage of virus to cohabitants introduced soon after the primary injection exposure (1wpc) provides evidence that natural shedding might be minimal in this early period of infection as PRV L1 RNA could not be detected in sentinel fish after 4 weeks of cohabitation. Even after 8 weeks of cohabitation, when 5 of the 6 sampled fish were positive for PRV L1 RNA, the systemic loads were still relatively low (<1e^3^ copies per µg RNA) in all but one fish (Fig. 6A); suggesting an early stage of dissemination.

PRV passage via cohabitation was successful during late stage infections. At least a portion of naïve cohabitants introduced at either 9 or 15 wpc became infected during 8 weeks of cohabitation (Fig. 6B,C). However, these infections tended to be slow to develop and more fish became infected when introduced at 9 wpc (7/12) than when introduced at 15 wpc (3/12). This might be a result of less infectious virus being shed in chronically infected fish than from fish at or near peak infection. Nevertheless, passage of virus during this late persistent phase was readily accomplished via i.p. injection of blood homogenate into naïve recipients which generated high-load systemic infections in 100% of fish within 4 wpc. Indeed, injection of this late-stage material into naive recipients yielded infection dynamics nearly identical to the first challenge with PRV 16-005ND, which had been harvested for passage at 4 wpc just when peak loads had been reached (Fig. 6B,C).

### PRV sourced from cohorts with and without HSMI-like lesions cause transient erythrocytic inclusions but not anemia in naïve recipients

**Figure 7:**
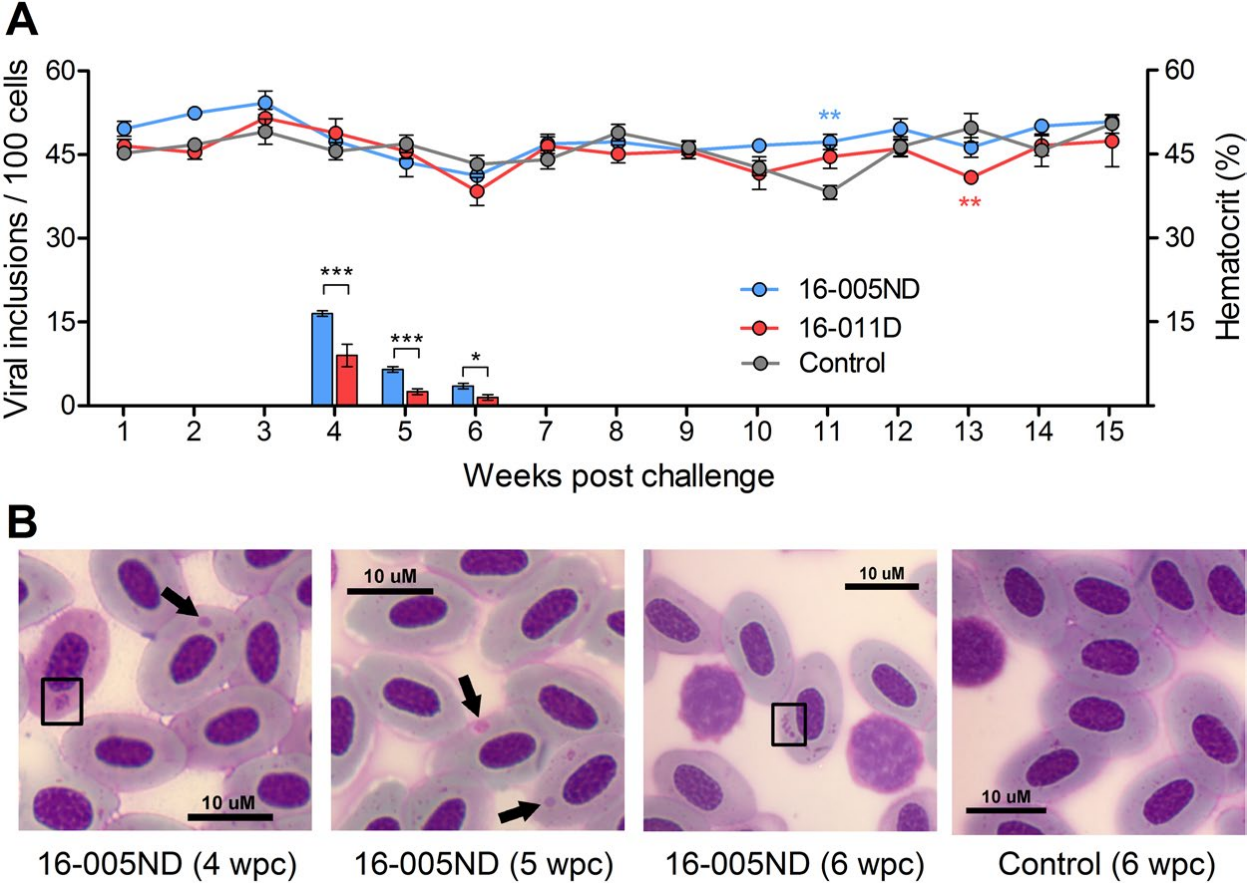
PRV sourced from blood of cohorts with and without HSMI-like lesions cause transient erythrocytic inclusions but not anemia in naïve recipients. (**A**) The trend in mean (±SEM) hematocrit (lines) for both PRV 16-005ND and 16-011D challenged fish in comparison to time-matched experimental controls did not suggest anemia in any treatment group. Erythrocytes with viral inclusions (bars) were observed between 4 and 6 wpc and were more prevalent in 16-005ND challenged fish (*p < 0.05; **p < 0.01; ***p < 0.001). (**B**) Erythrocyte cytoplasmic inclusions were defined as either a single, large spherical body (arrows) or as a cluster of smaller globular bodies (boxed) with moderate to dark staining.

Despite PRV reaching extreme (>10^9^ PRV L1 copies per mL) systemic blood loads following PRV 16-005ND and 16-011D injection challenges, neither PRV 16-005ND nor 16-011D caused a notable reduction in hematocrit (Fig. 7A). Relative to time matched controls, significant differences in hematocrit were uncommon (one occasion during each challenge) and inconsistent. Hematocrit was greater at 11 wpc in the PRV 16-005ND challenged group and less at 13 wpc in the PRV 16-011D challenged group and, in both instances, did not deviate beyond the range of control fish (38–50%). All values were well above an approximate 25% hematocrit threshold previously estimated to represent functional anemia in salmon^31^.

Transient viral inclusion bodies were observed by pinacyanol chloride staining between four and six weeks post challenge in blood smears of both PRV 16-005ND and 16-011D infected fish. Up to 15% of erythrocytes had inclusions and fish challenged with PRV 16-005ND had slightly higher quantities compared to those infected with 16-011D (Fig. 7A). Single, large spherical viral inclusions occurred at 4, 5, and 6 wpc; however, at 4 wpc when inclusions were first identified, affected erythrocytes from both challenge groups also had high prevalence of clusters of smaller inclusions. These primarily occurred in darkly stained (presumably immature) erythrocytes (Fig. 7B). At 5 wpc, large spherical inclusions predominated. By 6 wpc, inclusions appeared to be breaking up, as clusters of smaller inclusions again became dominant. No viral inclusions were observed at any other time point during these challenge trials.

### Systemic host transcriptional responses against PRV are minor, transient, and possibly load dependent

**Figure 8:**
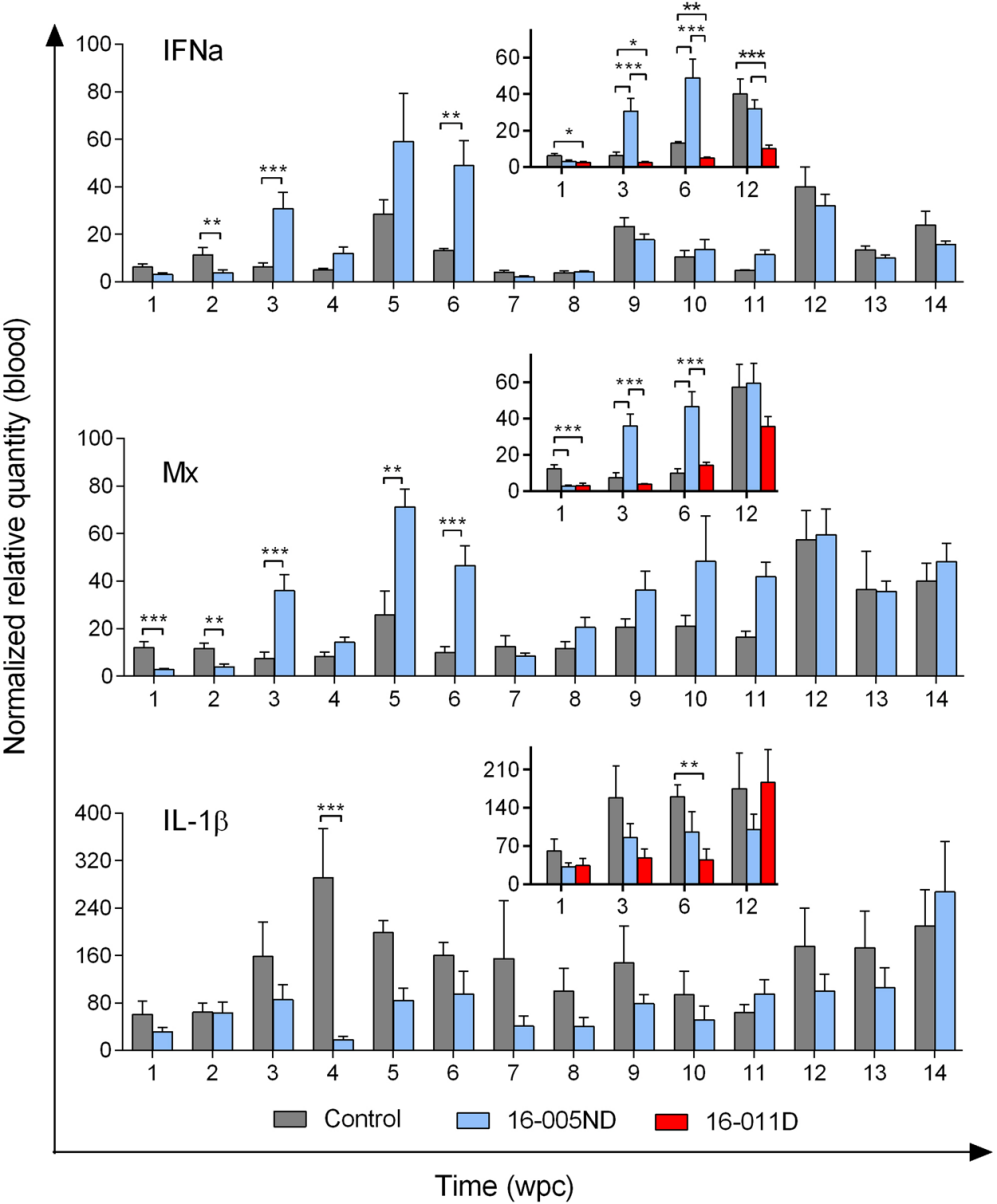
Systemic host transcriptional responses in blood against PRV are minor, transient, and possibly load dependent. The gene expression of *IFNa*, *Mx*, and *IL-1β* (proteins involved in viral response, antiviral, and inflammatory pathways, respectively) were monitored in 16-005ND challenged and control fish at weekly intervals and in 16-011D challenged fish at 1, 3, 6, and 12 wpc (see insert graphs). Significant (*p < 0.05; **p < 0.01; ***p < 0.001) increased expression from time-matched controls of *IFNa* and *Mx* but not *IL-1β* occurred when PRV 16-005ND load was near its peak (3-6 wpc). In each instance, expression was normalized to the stable expression of β-actin and scaled to the minimum observed value.

To identify if and when PRV is recognized by host blood cells, transcription of two classic viral recognition/antiviral defense genes were monitored during the PRV challenge trials: (i) *IFNa*, a type-I interferon of salmon which is generated in response to viral recognition in nearly all cell types^32^, and (ii) *Mx*, an antiviral response element triggered by cellular recognition of interferon^33^. Both genes were transcriptionally down-regulated during early infection (1-2 wpc) in both PRV 16-005ND and 16-011D infected fish relative to time-matched controls; however, as PRV infections progressed, significant induction of both *IFNa* and *Mx* occurred in 16-005ND but not 16-011D challenged fish (approximately 4-5 fold) when virus reached at or near peak loads (3-6 wpc) (Fig. 8). Neither *IFNa* nor *Mx* transcription was significantly up-regulated following 16-011D challenge at any of the time points analyzed. The lower systemic viral loads generated during peak 16-011D infection provides evidence that the response observed in 16-005ND was possibly load dependent. No significant change in *IFNa* or *Mx* transcriptional expression occurred during the late stages of PRV infection (7-15 wpc) in either 16-005ND or 16-011D challenges relative to time-matched controls. Further, monitoring of *IL-1β* transcription, an important cytokine involved in the inflammatory process of vertebrate and non-vertebrate animals including salmon^34^, also did not demonstrate significant systemic induction in blood of either PRV 16-005ND or 16-011D infected fish at any time point following challenge (Fig. 8). Rather, if any change occurred, it appeared that *IL-1β* transcription was slightly reduced in some instances relative to controls.

### Minor heart inflammation occurs during persistent late-stage PRV infections independent of donor fish disease status

**Figure 9:**
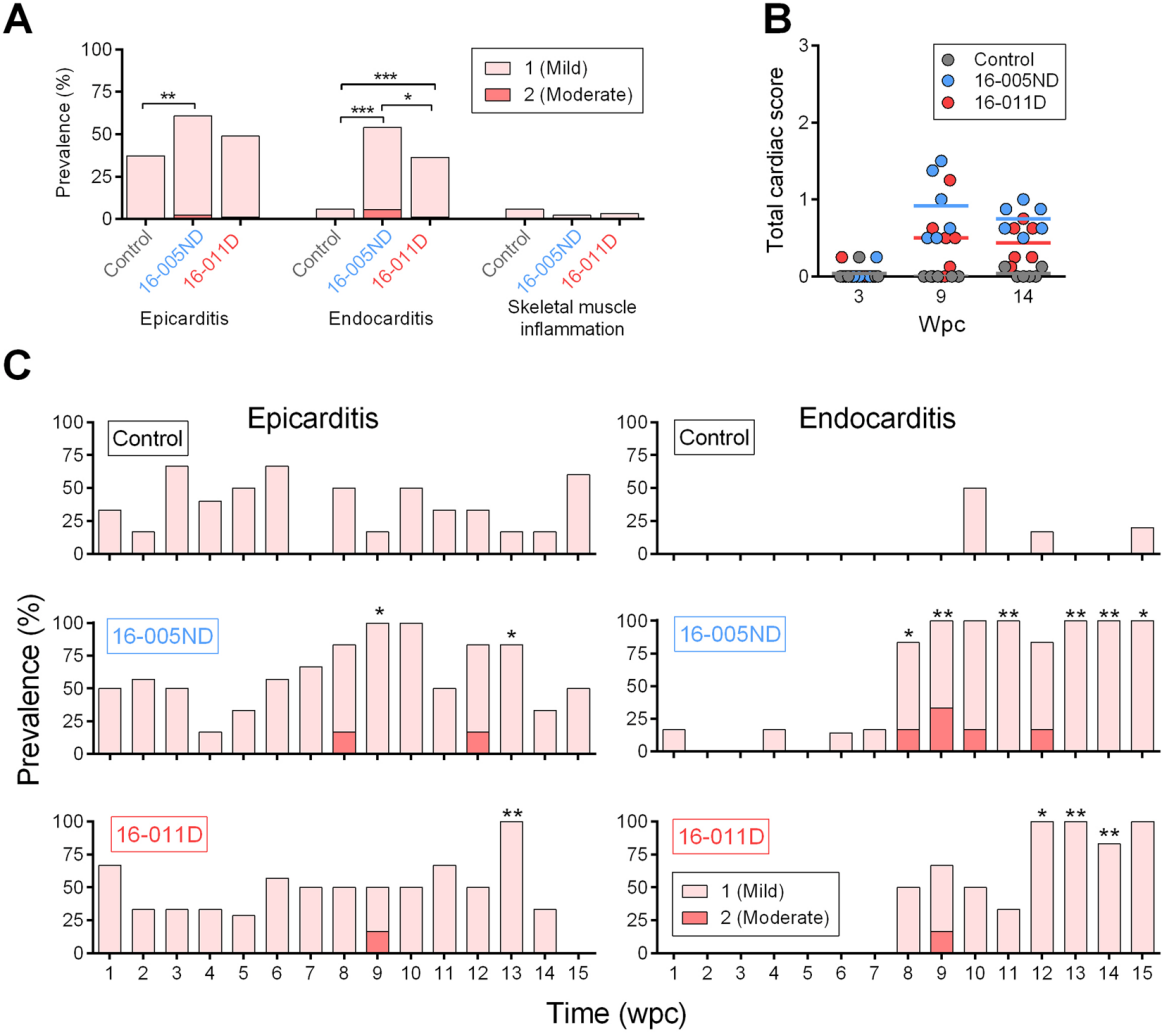
Minor heart inflammation occurs during persistent late-stage PRV infections independent of donor fish disease status. (**A**) The cumulative prevalence of epicarditis, endocarditis and skeletal muscle lesions identify significantly increased prevalence of heart but not skeletal muscle lesions in PRV challenged fish within 15 wpc (*p < 0.05; **p < 0.01, ***p < 0.001). However, **(B)** the overall heart severity scores (combined mean inflammatory score for the atrium, epicardium, compactum and spongiosa) did not progress beyond a mean value of 1 (mild) in any treatment group at 3, 9, or 14 wpc. **(C)** Prevalence and severity of heart lesions assessed at 7 day increments throughout the trial (n = 6 per time point) also identified mild epicarditis and endocarditis which at some time points were significantly elevated relative to controls. The occasional occurrence of lesions of moderate severity was only observed in PRV infected groups.

All heart as well as red and white skeletal muscle samples collected during this study were examined by pathologists GDM and/or HNS. A subset collected at 3, 9 and 14 wpc were also evaluated by a reviewing pathologist (Renate Johansen, Pharmaq Analytiq). In all instances, pathologists were blinded to PRV exposure status and lesions were scored according to severity (0 – none, 1 – mild/small amount, 2 – moderate, or 3 – severe/abundant) similar to methods applied in previous HSMI studies^1,8,22,35^ (Supplement 1). Mild lymphohistiocytic epicarditis was common in hearts of control fish during this study with a mean prevalence of 37% (±5% SEM) over the 15 week trial. Nevertheless, the prevalence of epicardial inflammation was greater in PRV 16-005ND challenged fish (mean 60 ± 5% SEM) which was accompanied by a significant increase in the overall median endocarditis severity score relative to controls (Fig. 9A). The prevalence of epicarditis in 16-011D challenged fish was also nominally greater than in controls (mean 48 ± 6% SEM), but the change in median severity score was not significant.

Endocardial tissues provided a clearer association between PRV and heart inflammation, where both 16-005ND and 16-011D challenged fish had significantly greater median severity scores for lymphohistiocytic endocarditis compared to controls (Fig. 9A). This mainly occurred between 8 and 15 wpc in both challenge trials representing the post-peak persistent phase of PRV infection within these populations (Fig. 9C). PRV was not associated with skeletal muscle inflammation, and of the 10 (out of 270) fish with mild skeletal muscle inflammation (all cases were mild), 5 were from control, 2 were from 16-005ND and 3 were from 16-011D challenged populations.

**Figure 10:**
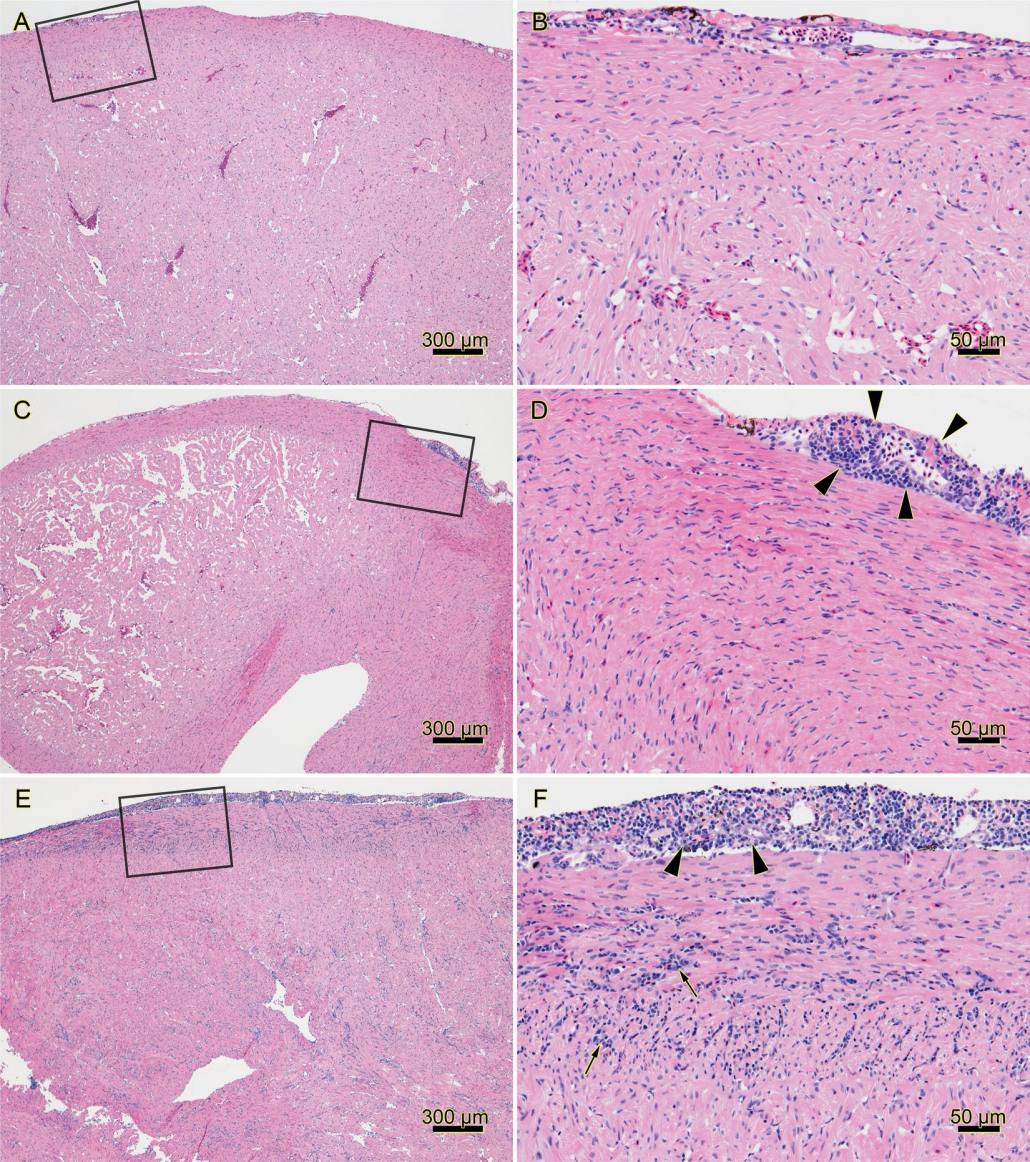
Complete range of histopathology in PRV 16-005ND and 16-011D infected fish encompassed no, low and moderate heart inflammation. Histopathology of hearts from PRV-injected and control fish at 8 week post-challenge identified (**A**,**B**) hearts with no microscopic lesions such as in control fish #184, (**C**,**D**) hearts with mild, focal, lymphohistiocytic epicarditis (arrowheads) such as in 16-011D challenged fish #191, or (**E**,**F**) hearts with either moderate lymphohistiocytic epicarditis (arrowheads), and/or endocarditis (arrows) such as from 16-005ND challenged fish #187. Note that fish #187 was the only fish in this study which had both moderate lymphohistiocytic epicarditis and endocarditis. Black boxes in the left column images outline the area shown at higher magnification in right column images; hematoxylin and eosin stain.

**Figure 11:**
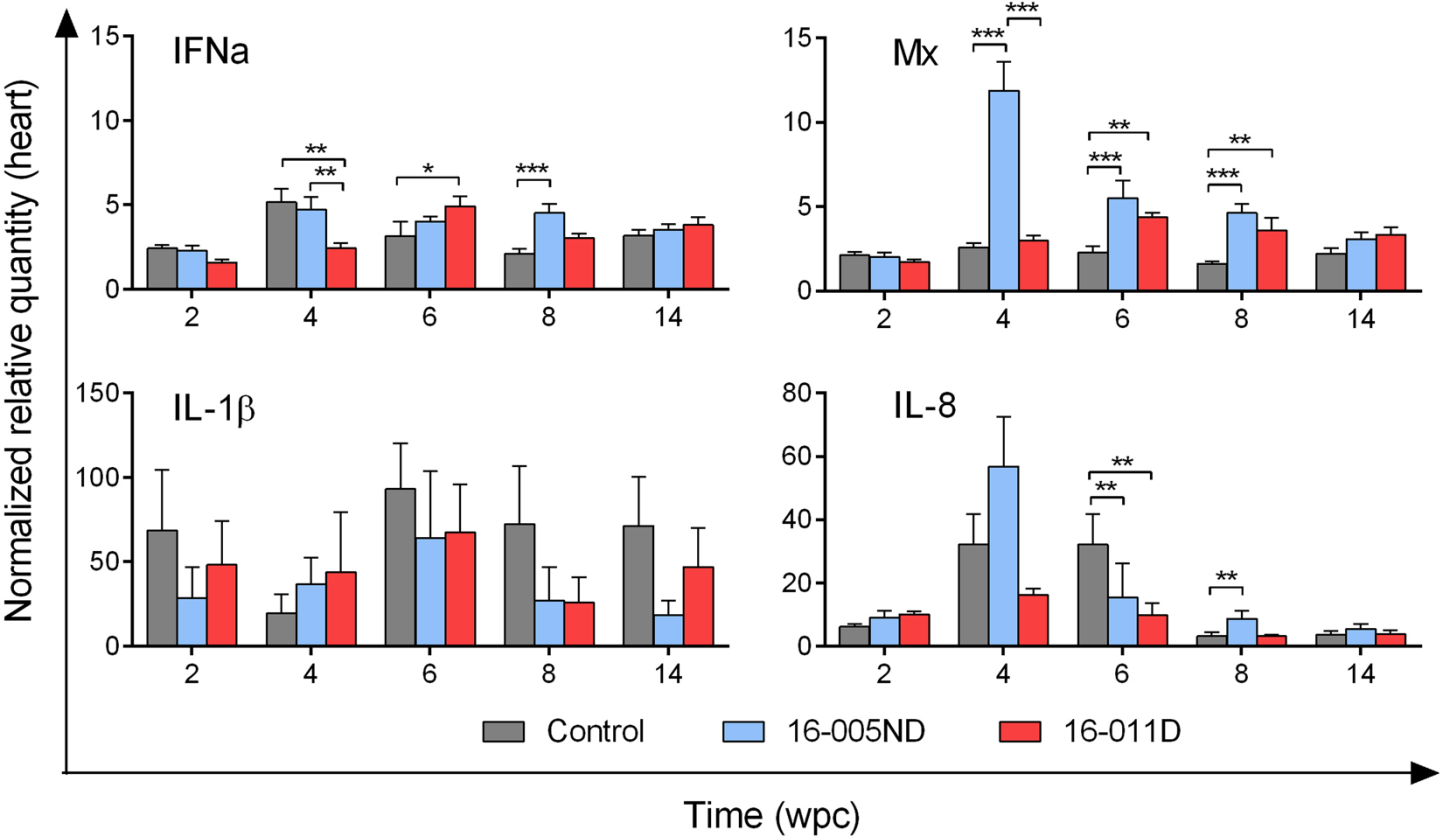
Minor and transient antiviral but not inflammatory gene transcription is induced in heart tissues following PRV infection. The transcriptional expression of *IFNa* and *Mx*, two classic viral response elements, had minor and transient up-regulation in response to PRV. Neither the inflammatory cytokine *IL-1β* nor the inflammatory chemokine *IL-8* had biologically relevant (> twofold) up-regulation within PRV infected heart tissues at the time points analyzed. Gene expression was normalized to the stable expression of β-actin and scaled to the minimum observed value.

Interestingly, fish challenged with PRV 16-005ND (which came from a non-diseased population) developed greater prevalence of heart inflammation than fish challenged with PRV 16-011D (sourced from fish with subclinical HSMI-like disease) (Fig. 9A). However, the overall severity of heart inflammation was generally mild in both PRV challenges (Fig. 9B), with typical heart tissues having no evidence of inflammatory lesions or only minor foci of inflammation (Fig. 10A–D). Indeed, the overall severity of heart lesions in PRV challenged fish (combined mean inflammatory score for the atrium, epicardium, compactum and spongiosa) was minor (mean score 0.9) even during the period of approximate highest prevalence and severity (9 wpc), which is below the severity threshold previously used to categorize an HSMI disease state (minimum total heart severity score > 1.5–2)^11^. One fish sampled during this study at 8 wpc (a PRV 16-005ND challenged fish) had both moderate epicarditis and endocarditis within the ventricle (Fig. 10E,F). However, even in this most extreme instance, the severity had not yet progressed into notable myocardial necrosis. The relatively minor impact of inflammation within heart tissues during this trial was further supported by a general lack of transcriptional induction for the inflammatory cytokine *IL-1β* or the inflammatory chemokine *IL-8* within PRV infected hearts (Fig. 11). Also, mild but significant antiviral responsiveness in heart tissues as demonstrated by *Mx* transcription was observed in response to both 16-005ND and 16-011D PRV challenge during the late-peak/early-persistent phase of infection; however, this only partially overlapped the timing of heart inflammation.

## Discussion

First identified in 2010, PRV represents the newest member assigned to the *Orthoreovirus* genus^18^. Although Orthoreoviruses are almost certainly the most well-studied within the *Reoviridae* family, there is considerable diversity in pathogenicity and disease association within the genus for which much is still unknown^36^. PRV adds yet further complexity by associating functional and genetic commonalities to both the Aquareoviruses and Orthoreoviruses while remaining phylogenetic and functionally distinct^37,38^.

In Norway, most commercial Atlantic salmon become PRV positive, but only some develop HSMI. This does not appear to be dependent on systemic PRV load, and it is not clear why some farms experience high losses due to HSMI while others do not. Nevertheless, clinical outbreaks of HSMI in farmed Atlantic salmon of Norway are reasonably common^10,11,18,39^, and laboratory challenge trials have demonstrated a clear ability for PRV to cause severe heart lesions^1^. Indeed, laboratory challenges trials in Norway routinely generate severe heart lesions in accompaniment with occasional skeletal muscle lesions that are similar to lesions observed in diseased salmon on farms^11,19–21,40^.

The results of our study expand our understanding of a strikingly divergent relationship regarding PRV and its association with disease in Pacific Canada. PRV also appears to be highly prevalent in farmed Atlantic salmon of Pacific Canada^5^; yet, only rare subclinical cases of farm-level HSMI-like pathology have been reported and a clinical outbreak of HSMI as described in Norway^10,11^ has never been described. Here we diagnosed one case of an HSMI-like disease state from a pre-transfer government farm site audit, with up to five additional cases being identified between 2011 and 2013^7,8^. Even if all isolated cases of idiopathic cardiopathy observed during these audits were presumed to be HSMI, it would constitute approximate 2% prevalence within dead and dying farmed Atlantic salmon of British Columbia. Because annual mortality among farmed Atlantic salmon in Pacific Canada is about 10–15%, HSMI-like lesions would thus (at most) be associated with only about 0.3% annual mortality across the industry. In a laboratory setting, PRV from Pacific Canada has failed to cause severe heart lesions or any severity of skeletal muscle inflammation despite establishing high-load blood infections^13^. Here we confirm these findings using PRV from two different commercial sources in Pacific Canada which similarly replicated to high loads following experimental infection.

The consistent dissimilarity in disease outcome following PRV infection in Atlantic salmon of Norway versus Pacific Canada leads to the rather straightforward hypothesis that something within the host-pathogen-environment dynamic is different between these two geographic regions. One potential difference is that genetic divergence in PRV between these two regions is sufficient to result in altered virulence. The sequencing of PRV genomic material from two discrete sources in this study supports previous observations that PRV appears to have low phylogenetic diversity within farmed Atlantic salmon of Pacific Canada yet is relatively distinct from Norwegian isolates^14,41^ (Fig. 2B).

Small genomic changes can be enough to drastically alter the virulence of a virus, such as in the HPR0 variant within infectious salmon anemia virus (ISAV)^42^ or VP2 variants of infectious pancreatic necrosis virus (IPNV)^43^, and it is possible that one or more of the three putative amino acid changes unique to the PRV isolated from non-diseased fish used in this study could be held responsible for an increase in virulence. However, as pointed out previously^13^, altered virulence associated with genetic viral variation is almost always accompanied by distinct tropisms and/or altered replication kinetics. For example, HPR0 variants of ISAV have altered tissue tropisms (gill specific rather than systemic)^44^ and low-virulent IPNV variants are correlated with reduced *in vivo* loads compared to high virulence stains^45^. Similar differential tropisms or replication kinetics do not appear to be evident when comparing PRV sourced from cohorts with (16-011D) and without (16-005ND) HSMI-like lesions in our study. Further, HSMI-like disease could not be passed from field-infected fish into a virtually identical cohort of Atlantic salmon held in a laboratory setting. We therefore hypothesize that at least in Pacific Canada, the ability for PRV to cause HSMI or any form of severe heart lesions cannot be attributed to a genetic variation of the virus alone.

It is nevertheless possible that the previously reported instances of HSMI-like disease in Pacific Canada, although sharing similar microscopic lesions, were not generated by the same mechanism(s) as in Norway. For example, perhaps PRV was contributing to and exacerbating HSMI-like disease in farmed Atlantic salmon of Pacific Canada that was initiated by alternate or synergistic factors. By this reasoning, any of the 27 putative amino acid changes observed in this study between the Canadian and Norwegian PRV variants could act alone or in concert to produce an altered state of virulence responsible for the rather striking prevalence discrepancy for HSMI in these two countries. It therefore becomes important to consider the phenotypic characteristics of PRV following analogous laboratory challenge trials conducted in Norway and Canada which may support this hypothesis.

**Figure 12:**
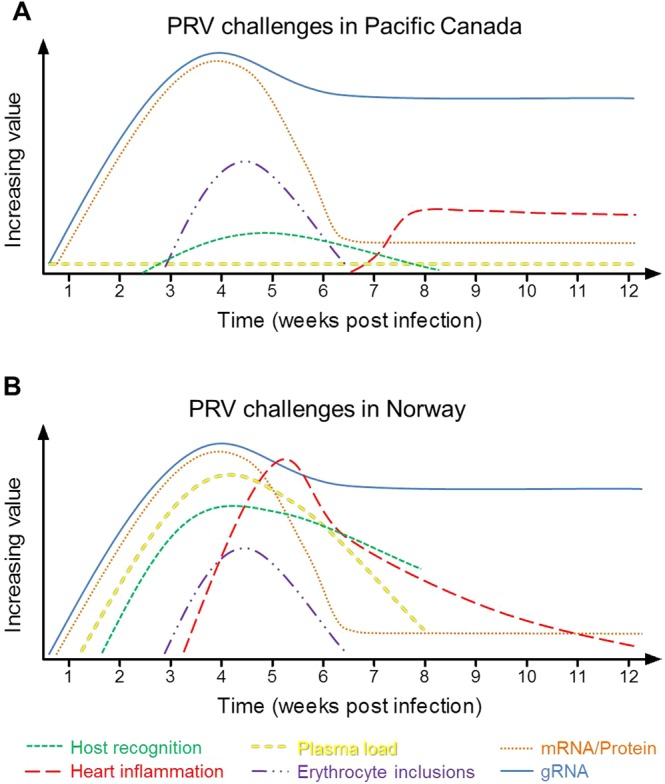
Contrast summary for trends in PRV phenotypic infection dynamics between Norway and Canada laboratory challenge of Atlantic salmon. In comparing the present challenge trials conducted in (**A**) Pacific Canada with results from similar challenge trials conducted in (**B**) Norway^1,20,21,40,45^, the kinetics of viral RNA, protein (indirectly measured in Canada by mRNA or directly measured in Norway by florescent antibody staining) and erythrocytic inclusion body formation follow a similar pattern. However, the kinetics regarding the plasma load of PRV, transcriptional induction of genes involved in innate host recognition of virus by Atlantic salmon, as well as the severity and timing of PRV associated heart inflammation appear notably discrete between the two countries. For these comparisons, timing is presented relative to first signs of infection at 10-12 °C and not necessarily the initiation of a challenge since detectable infections take longer to develop following cohabitation than by i.p. injection. It should also be noted that no data are yet available with regard to the transcriptional host innate immune responses or plasma PRV loads beyond 8 weeks post infection in Norwegian based studies and thus their late stage kinetics are unknown and not presented. Lastly, Y-axis scale is not intended to be interpreted as absolute.

The phenotypic characteristics of both Canadian and Norwegian PRV during infection have at least three potentially significant dissimilarities (Fig. 12). First, the inability to detect Pacific Canada PRV (either 16-005ND or 16-011D) in the blood plasma of infected fish during our challenges is in stark contrast with PRV loads reported in plasma of infected fish following challenges with Norwegian PRV, where sometimes substantial PRV plasma loads are generated for a period lasting at least six weeks (2 to ≥8 weeks following detectable infections)^1,40^. Second, there is a considerable difference in scale regarding host recognition of PRV. Although direct comparisons between Canadian and Norwegian studies are limited because Canadian studies have assessed gene expression relative to time-matched controls whereas Norwegian studies have referenced expression to time zero, it is nevertheless conspicuous that mean systemic and heart-specific antiviral responses increased no more than fivefold in our study whereas in Norwegian challenges these genes increased 10–50 fold in the blood^1,46^ and more than 100 fold in the heart^21^. The comparative lack of antiviral response to Pacific Canada PRV compared to Norwegian PRV is further supported by the relative protection PRV has afforded to fish challenged with a secondary virus (IHNV) in Norway^47^ but not in Pacific Canada^28^. Lastly, in addition to the discrepancies concerning the severity of heart inflammation, the timing of PRV associated heart inflammation is also different between challenges conducted with PRV from these two countries. Specifically, by either injection or cohabitation exposure of PRV, heart inflammation (prevalence and severity) in Norwegian studies consistently begins around the time of peak systemic PRV load, reaches high severity 1-2 weeks later, and thereafter diminishes^1,20^. In contrast, increased prevalence of heart inflammation in our challenge trials did not occur until approximately 4 weeks after peak PRV systemic loads were reached and maintained high prevalence (although not severity) for the remaining 6-7 weeks of the trial.

Taken together, these findings suggest that phenotypic differences in infection kinetics between PRV from Norway and Pacific Canada stem at least in part from genetic variation between the two regional variants. Although a specific virulence factor (or set of factors) remains unclear, we hypothesize that the increased prevalence of Norwegian PRV outside red blood cells (i.e., in the plasma) might help to explain the heightened recognition of PRV by Norwegian fish and that this recognition could contribute to the elevated inflammatory immune responses observed. Because the S1 segment is responsible for the spread of mammalian orthoreovirus^48^ and also has high genetic diversity between Norwegian and Canadian PRV^14^, it presents a likely candidate for further investigation as a virulence factor for PRV. However, the involvement of cytotoxic T-cells and a robust type-II interferon response generated in heart tissues of HSMI diseased Atlantic salmon in Norway^21^ almost certainly enhances or even causes the observed tissue damage. PRV from Pacific Canada also elicits a specific cytotoxic T-cell response in heart tissues of Pacific-adapted Mowi-McConnell Atlantic salmon, but to far less of a degree^49^. This presents the possibility that HSMI might result (or be enhanced) by a host-associated hypersensitivity heightened in the Norwegian-adapted Atlantic salmon farmed in Norway relative to the Pacific-adapted Mowi-McConnell strain farmed in Pacific Canada. This hypothesis is supported by the development of an HSMI resistant strain of Atlantic salmon in Norway that is somewhat resistant to disease but not PRV infection^50,51^. Ultimately we believe that both host and virus specific factors likely play a role in the development of HSMI, and further investigations are needed to pinpoint the mechanisms responsible for generating this disease.

The kinetics of North American PRV in Atlantic salmon as observed in this study indicates three distinct phases of infection: early, peak, and persistence. In the first (early) phase of infection which lasts 2-3 weeks at 10-12 °C, initial replication and dissemination of the virus into the blood cells of the host occurs. During this period, viral ‘factories’ (inclusions) were not observed in erythrocytes and there seemed to be no systemic recognition of virus by host cells. This indicates that the virus might be replicating by an alternative process relative to later time points or it might be initially infecting a different cell type. Mammalian orthoreoviruses first infect epithelial cells of the small intestine or lung prior to hematogenous dissemination^52^; and the recent detection of PRV in intestinal enterocytes^7^ indicates that a similar course of infection might be followed by PRV. Regardless, it is expected that this early replicative phase dictates the overall severity of infection^53^, as it likely accounts for how many erythrocytes ultimately become infected. This is supported by the discrepancy in total virus production following challenge with PRV 16-005ND compared to 16-011D in this study, where an initial lag in 16-011D replication appeared to be the major difference between otherwise identical replication dynamics within the two challenges. What caused  this poor primary infection rate following challenge with PRV 16-011D compared to 16-005ND is unclear; however, the lack of PRV transmission via fish cohabitation at this early stage of infection suggests that whatever cell type the virus is infecting during this period, it is not likely being shed into the environment to a high degree.

In the second (peak) phase of infection that lasts 2-3 weeks  at 10-12 °C, substantial PRV replication within erythrocytes occurs along with the formation of cytoplasmic viral inclusions. The large spherical inclusions observed here were similar to those previously reported in Norway^40,46,54^ as well as to those that develop during mammalian reovirus infection of well-established cell lines^55^. The highest systemic loads of PRV RNA occur during this period; which appeared sufficient in some instances to initiate mild systemic host recognition of virus. As host recognition was only observed following 16-005ND challenge and not following PRV 16-011D challenge in this study, we speculate that this recognition was load dependent since PRV 16-005ND reached higher transcriptional quantities during this period. From previous cohabitation challenges, it is also concluded that substantial shedding of virus occurs at this time^13^.

In the third (persistent) phase of infection, viral inclusions within erythrocytes disappear. No systemic host recognition of virus occurs, and a marked reduction in viral protein production (as measured by mRNA concentration) occurs even though large quantities of genomic PRV material remain associated with the erythrocyte cell fraction. This supports the hypothesis that infectious PRV is retained in the cytoplasm of infected erythrocytes but is in a reduced or non-replicative state. The ability to recapitulate infectious replication of PRV from this late stage of infection was readily accomplished by injecting lysed blood cell material into naïve fish which generated comparable temporal infection dynamics to virus that had been harvested during the peak phase of infection (Fig. 6). However, poor viral transmission occurred via cohabitation during this late infectious stage, suggesting natural shedding of virus might be minimal during persistent infections and may even cease entirely over time. This is supported by the reduced rates of infection by cohabitation at 15 wpc compared to 9 wpc in this study, and the inability to transmit virus via cohabitation after 45 or 63 wpc as demonstrated previously^13^. Nevertheless, infectious virus was still present in the blood of infected fish in this study for at least 15 wpc and it is presumed that the rather substantial quantities of RNA detected in blood at 63 wpc by Garver *et al*.^13^ also represented at least a moderate amount of infectious PRV particles.

For this study, we focused mainly on PRV L1 RNA to monitor viral loads in accordance with a number of previously published works^13,18,28,56–58^. However, some studies have targeted alternate segments of the PRV genome for relative quantification. Specifically, Haatveit *et al*. identified differential temporal expression of PRV S1 compared to M2 and M3 during the persistent phase of infection in a Norwegian challenge trial, where the relative quantities of S1 were approximately 9 Ct less than M2 and M3 (a >500 fold theoretical reduction)^46^. In our study, the relative quantity of all 10 genomic segments was comparable during all three phases of infection with less than fourfold proportional divergence between any two given segments (Fig. 5). This indicates that for the Pacific Canada PRV we tested, any of the 10 segments could be used interchangeably to estimate PRV abundance within a sample at any given time. In Norwegain based studies, temporal expression patterns of L1^20^ as well as M2 and M3^46^ appear similar to what has been observed here; however, the considerable reduction in S1 expression observed by Haatveit *et al*.^46^ provides yet further support for altered viral kinetics between Norwegian and Pacific Canadian strains of PRV that may stem from alterations in the S1 segment of the genome.

In addition to exploring the expression patterns of all 10 PRV genomic segments, we also developed a new and relatively simple technique for differentiating the amount of PRV single-stranded mRNA from double-stranded genomic material. It is likely that this technique can be applied in the detection of RNA from any virus with a dsRNA genome which might have expanded application in the laboratory exploration of dsRNA viruses. In specific context to PRV, we observed that the quantity of mRNA (and by implication the quantity of new viral proteins being made) became significantly reduced during the final persistent phase of infection. This is in line with previous observations of λ1, μ1, σ1 and σ3 PRV protein production as noted in late stage infections of Atlantic salmon in Norway^46^. We also observed relatively minor but statistically significant proportional differences of mRNA quantities for individual PRV genomic segments which varied depending on the phase of infection. However, the implications for this latter variability is unknown and could be inconsequential given that almost all variation was encompassed within a twofold deviation from complete proportional equality.

One important aspect of the host immune response to PRV that was not addressed in this study was the putative development of antibodies. In Norway, host Atlantic salmon have been demonstrated to generate detectable antibodies specific to PRV µ1c and µNS in the plasma that began approximately two weeks after peak systemic PRV loads were reached and were maintained at detectable levels until the end of the study one month later^59^. It is unknown as to whether antibodies were generated against PRV in our current study; however, given the high viral loads, relative ease for horizontal transmission, and mild but significant innate antiviral recognition observed during peak PRV infections in this study, we speculate that at least some PRV specific antibodies were produced. If so, there may be future potential in exploring the avirulent characteristics of the Pacific Canada PRV used here to potentially protect against Norwegian isolates of PRV that have been associated with causing HSMI, particularly since both formalin killed PRV and DNA plasimids expressing PRV µNS have demonstrated at least partial protection against HSMI in Norway^22,60^.

In conclusion, although we were able to identify an HSMI-like disease state in farmed Atlantic salmon of Pacific Canada, this regionally rare condition could not be effectively transmitted via injection of PRV infected blood material into naïve fish as has been accomplished in Norway. This study also revealed genotypic and phenotypic differences between PRV from Pacific Canada compared to what has been reported from PRV challenge trials in Norway. These differences suggest that virus and/or host specific factors are likely needed for the development of HSMI in farmed Atlantic salmon and that currently PRV infections of Atlantic salmon in Pacific Canada are of low virulence. Further research is needed to determine the potential cause or causes for HSMI-like lesions in Pacific Canada. Also, because interactions between Atlantic salmon, PRV, and the environmental conditions of Pacific Canada do not appear conducive to HSMI development, the low virulence associated with Pacific Canada PRV provides a useful model for comparative studies to investigate the requirements for initiating PRV-associated disease and exploring possible protection mechanisms against HSMI.

## Methods

### Fish source and husbandry

Atlantic salmon for the challenge studies were sourced from a single commercial freshwater hatchery on Vancouver Island, British Columbia and brought to the Pacific Biologic Station (PBS) in Nanaimo, British Columbia, Canada. Pre-transport screening of 20 fish via qPCR was negative for PRV. Tissue homogenates from 90 fish in the hatchery were negative for culturable agents; no cytopathic effect was observed on CHSE or EPC cell lines prior to transport. Fish were of a Pacific-adapted Mowi-McConnell strain of Atlantic salmon with at least 30 years isolation from the originating European stocks^27^. Once at PBS, fish were maintained in UV treated municipal freshwater (10° ± 1 °C) for 3 months prior to smoltification to undiluted sand-filtered UV-irradiated seawater (11° ± 1 °C, 32 ppt). A natural photoperiod was used during culture and fish were fed dry pellets (EWOS) at 1-2% body weight per day prior to challenge. This cohort was used as a source for PRV negative control inoculum, provided the naïve recipients for the primary PRV 16-005ND and 16-011D i.p. injection challenges, and also provided the naïve recipients for the subsequent i.p. and cohabitation viral passage experiments using PRV 16-005ND. During all challenge trials, fish were maintained on undiluted UV-irradiated seawater (11° ± 1 °C, 32 ppt) and fed a ration of EWOS pellets at 1% body weight per day.

### PRV detection and quantification

#### PRV (L1) detection by TaqMan qPCR

PRV nucleic acid was detected from blood, heart and plasma samples by real-time qPCR with slight modification to previously described methods^13,28^. In summary, total RNA was extracted from 100 μL blood, 100 μL plasma, or ~50 mg heart tissue in TRIzol Reagent (Life Technologies) as per manufacturer’s instructions using 5 mm steel beads and TissueLyser II (Qiagen) which operated for 2 min at 25 Hz. A portion of eluted RNA (1.0 μg) was denatured for 5 min at 95 °C, immediately cooled to 4 °C, and reverse-transcribed using a High Capacity cDNA Reverse Transcription kit (Life Technologies) following the manufacturer’s instructions. Resulting cDNA was used directly as template for qPCR analysis in a StepOne-Plus real-time detection system (Applied Biosystems) using primers and TaqMan probe targeting the L1 fragment of the PRV genome^28^. Each reaction contained 400 nM primers and 300 nM TaqMan probe, 1X TaqMan Universal Master Mix and 1 μL cDNA template within each 15 μL reaction. Cycling conditions included an initial incubation of 95 °C for 10 min followed by 40 cycles of 95 °C for 10 s and 60 °C for 20 s. Samples were assayed in duplicate and were considered positive if both technical replicates reported a Ct value < 40 cycles. Absolute PRV quantification was determined in each instance by serial dilution of a 482 bp double-stranded DNA gBLOCK fragment (Integrated DNA Technologies) consisting of sequence targeted by the qPCR primer and probe^13^. A seven-step 10-fold dilution series of the gBLOCK fragment spanning a dynamic range of 10–10^7^ target copies per reaction was incorporated in duplicate into each run. The limit for accurate quantitative assessment (< 20% CV) using this technique was calculated to be between 10–50 copies with a limit of detection (at >90% prevelence between replicates) of 1–3 copies per reaction determined as previously described^61^.

#### PRV (all segment) detection by SYBR^®^ green qPCR

Primers specific to each of the 10 PRV genome segments were designed using Primer3^62^ within the Geneious 9.1.7 software platform^63^ to homogenous protein-coding regions within four published PRV genomes previously identified from Pacific Canada: VT06062012-358^41^; BCJ19943_13^14^; and WSKFH12_14^14^ (Supplement 1). A portion (1 μL) of cDNA generated using a High Capacity Reverse Transcription kit from Trizol extracted RNA as described above was added to duplicate 15 μL qPCR reactions containing 500 nM forward and reverse primers and 1x final concentration of Power SYBR^®^ green PCR master mix (ThermoFisher) in molecular grade water. Reactions were analyzed in a StepOne-Plus real-time qPCR detection system (Applied Biosystems) with an initial polymerase activation at 95 °C for 10 min followed by 40 cycles 5 s at 95 °C, 20 s at 60 °C, and 10 s at 72 °C with fluorescence measured at the end of the 72 °C step. Melt curve analyses were performed to ensure amplification specificity and a five-step fourfold dilution series of cDNA prepared from the blood of a highly infected individual (sample #35; 16-005ND4 at 4 wpc) was performed in duplicate on each run to estimate relative quantity and amplification efficiency.

#### ssRNA PRV qPCR detection and validation

All TaqMan or SYBR qPCR analyses designed to exclusively amplify the ssRNA and not dsRNA component of the targeted PRV segment were performed as described above with the exception that RNA was heated to 80 °C for 1 min rather than 95 °C for 5 min prior to cDNA synthesis. Total RNA from PRV 16-005ND infected fish at 10 wpc was used to validate this differential detection of PRV ssRNA by exposing extracted RNA (2 µg) to either no enzyme, 2 U Pure Link™ RNAse A (ThermoFisher Scientific), or 2 U RNAse A in the presence of 0.5 M sodium chloride which selectively protects dsRNA but not ssRNA from RNAse A degradation^64^. Following incubation at 25 °C for 45 min, RNA (if remaining) was recovered using RNeasy MinElute Cleanup Kit (Qiagen) as per manufacturer’s instructions. Recovered RNA was heated to either 55 °C for 5 min, 80 °C for 1 min, or 95 °C for 5 min, immediately cooled to 4 °C and reverse transcribed using a High Capacity cDNA Reverse Transcription kit (Life Technologies) following the manufacturer’s guidlines. Resulting cDNA was used directly as template for PCR analysis in a StepOne-Plus detection system with 500 nM PRV L1 (this study) or Atlantic salmon β-actin^13^ forward and reverse primers and 1X Power SYBR^®^ Green PCR master mix (ThermoFisher). Cycling conditions were performed as described above but were ended after 30-cycles prior to fluorescence saturation in samples for which product was amplified. A portion (5 µL) of product was then visualized by UV excitation on a 3% agarose Tris-borate-EDTA (TBE) gel containing 0.5x SYBR^®^ Safe DNA stain after 45 min migration at 120 volts in 1X TBE running buffer (ThermoFisher).

### PRV sequencing

Library construction, sequencing services and bio-informatics support was provided by the Canadian Centre for Computational Genomics and Génome Québec Innovation Centre, Montréal, Canada. RNA extracted from the blood of four fish were selected for library construction and RNA-seq analysis – two from fish challenged with PRV 16-005ND (sample numbers 161 and 165; see Supplement 1) and two from fish challenged with PRV 16-011D (sample numbers 167 and 171) collected at 7 wpc (Fig. 2A). A portion (10 µg) of the total RNA extracted from each samples was purified using 2 U of DNase I (Life technologies) at 37 °C for 45 min followed by RNeasy MinElute Cleanup (Qiagen) as per manufacturer’s instructions. RNA quality was visualized on a 1% bleach denaturing gel^65^ and ensured to have a Bioanalyzer (Agilent) RNA Integrity Number (RIN) > 9. RNA sequencing was performed using half of one lane (8 libraries per lane) of an Illumina® HiSeq 2500 (Illumina Inc.) platform using a NEBNext^®^ rRNA Depletion Kit (Human/Mous/Rat) with read lengths of 125 bp. Base calls were made using the Illumina CASAVA pipeline encoded in Phred 33. The two libraries generated for both 16-005ND and 16-011D challenges were pooled and *de novo* transcript assembly was performed on combined reads (2 libraries per assembly) following the pipeline described by Haas *et al*.^66^ based on the Trinity assembly software suite^29^. In brief, reads were trimmed using Trimmomatic software^67^ from the 3′ end with a minimal Phred score of 30 and a minimum length of 32 bp. A normalized metric of reads was generated using Trinity normalization utility and surviving paired reads were assembled using the Trinity assembler^66^. Putative assembled transcripts were aligned against the NCBI Viral Genomes Resource database^68^ using the blastn program from the NCBI BLAST family. Transcripts which aligned to PRV sequences with an Expect value (E value) less than e^−100^ were aligned by segment using Geneious 9.1.7 to report the consensus sequence for the longest positive-sense genomic strand in each instance.

### PRV and HSMI sampling during natural infection

Moribund or recently deceased net-pen farmed Atlantic salmon were collected as part of a pretransfer fish health audit conducted by the Fisheries and Oceans Canada, Aquaculture Management Division. With relevance to this study, skeletal muscle and heart tissues were preserved in 10% neutral buffered formalin and processed as previously described^5^. On July 5^th^, 2016, an audit of 40 fish (36 moribund/dead; 4 live) was conducted at a net-pen farm site in the Johnstone Strait of BC, for which skeletal muscle and heart tissues were collected for histology. The site had 12 operational pens; each containing between 30–50 thousand fish per pen with fish weighing approximately 500 g each. On July 29^th^ and August 7^th^, 2016, samples of heart and skeletal muscle from 5 and 6 moribund/dead fish, respectively, were also collected for histology by a private veterinarian working for the source farm.

A final sampling was conducted August 19^th^, 2016 by MPP and KAG, in which 20 fish were sampled specifically for this study – six fish were moribund/fresh dead, eight were from the general population (apparently healthy), and six were non-performers of low body condition. Blood (0.5–3 mL) was collected from the caudle vein of each fish using 22 gauge needle and 3 mL syringe. A 100 µL subsample was immediately frozen in liquid nitrogen and used for PRV L1 TaqMan qPCR screening as described above. Remaining blood was divided into 1 mL aliquots and stored at −80 °C for use in generating challenge inoculating material described below. Heart and skeletal muscle from each fish was preserved in 10% neutral buffered formalin for histopathologic evaluation.

### PRV challenge of Atlantic salmon by i.p. injection

#### Inoculum preparation

PRV 16-005ND was initially sourced from a cohort of healthy Atlantic salmon in March of 2016 held at a commercial freshwater Atlantic salmon rearing facility on Vancouver Island, Canada. The facility had no history of HSMI and PRV material collected from this site in 2013 had failed to generate HSMI in previous laboratory challenge trials^13,28^. PRV infected blood of hatchery fish (~25 g) which had been frozen at −80 °C prior to use was passed through ~30 g British Columbia Mowi-McConnell Atlantic salmon held in brackish water (15ppt) for three weeks at 11 °C, passed again through ~50 g Atlantic salmon held in seawater (32 ppt) for three weeks at 11 °C, and passed a third time in ~55 g Atlantic salmon held in seawater for four weeks at 11 °C prior to final collection. In each instance, blood from three infected fish was pooled, diluted 1:10 in Hank’s balanced salt solution (HBSS), sonicated on ice for 80 s in 10 s bursts with 30 s rests using a Branson Digital Sonifier 250/450 at 20% amplitude, clarified via centrifugation at 2,000 × g for 5 min at 4 °C, and administered to naïve fish by 100 µL intra-peritoneal injection. Following the third passage, blood from three fish was pooled and inoculate prepared as for previous passages.

PRV 16-011D was sourced from the blood of three fish (#3, 5, 15) collected at the net-pen farm site in which fish had HSMI-like lesions on August 19^th^, 2016 (Supplement 1). The blood from these fish was pooled, diluted 1:10 in HBSS, sonified and clarified as described for 16-005ND. An identical preparation of sonified and clarified diluted blood was prepared from a pooled sample of three PRV-free individuals sourced from the cohort of fish used for all subsequent challenge trials to provide vehicular control inoculate.

#### Intra-peritoneal injection and monitoring

Atlantic salmon (~70 g each) were anesthetized in an aqueous solution of Tricaine methanesulfonate (0.05 g/L) and given a 200 µl intra-peritoneal injection of either PRV 16-005ND inoculum, PRV 16-011D inoculum, or PRV-free vehicular control inoculum (90 fish per treatment). Fish were placed in treatment-specific 850 L circular tanks (one tank per inoculum) supplied with 30 L/min 11 °C (±1 °C) UV-irradiated seawater (32 ppt). Temperature, dissolved oxygen, feeding performance, and morbidity/mortality were monitored daily throughout the challenge trials (Supplement 1).

#### PRV and HSMI associated sampling

Fish were anesthetized in an aqueous solution of Tricaine methanesulfonate (0.05 g/L), and blood and tissue samples were collected from six fish per treatment tank at 7 day intervals through 15 wpc. Blood (~2 mL) was collected using a 22 ga needle and 3 mL syringe. A 100 µL aliquot was immediately frozen in liquid nitrogen and subsequently used for PRV screening and gene expression analysis by qPCR as described above. Approximately 10 µL was transferred to a sodium-heparin treated Fisherbrand™ micro-hematocrit tube and spun at 15,000 × g for 10 min for hematocrit determination. A second 10 µL was smeared on a glass microscope for cytoplasmic inclusion body visualization. Slides were air dried, fixed in 100% methanol for 5 min, and stained with pinacyanol chloride^40^. Remaining blood was transferred to a heparinized vacutainer and spun at 2,000 × g for 5 min at 4 °C. Plasma (100 µL) was transferred to a clean 2 mL microtube and immediately frozen at −80 °C prior to PRV qPCR screening as described above. An approximate 200 mg section of red/white skeletal muscle was excised from the left lateral line at approximately the mid-body and preserved in 10% neutral buffered formalin for histopathology. Hearts were bisected longitudinally and one half preserved in 10% neutral buffered formalin for histopathology while the other half was immediately frozen in liquid nitrogen for qPCR analyses. Tissues in 10% NBF were fixed for 24–48 hours, transferred to 70% isopropanol and paraffin embedded following standard methods. Sections 3 µm thick were transferred to glass slides and stained routinely with haematoxylin and eosin for light microscopy^5^. Photomicrographs were optimized for illumination and color balance^69^. To ensure inter-sample consistency in lesion scoring as well as consistency relative to published PRV studies from Norway, approximately 10% of slides (29/338) were examined by both principal pathologists (GDM and HNS) and a subset of 60 slides was also sent to a third reviewing pathologist (RJ) in Norway for further examination (Supplement 1). All histopathology was conducted blind to PRV exposure status and scores provided by the other pathologists.

At 10 wpc PRV 16-005ND, blood of three fish was separated into plasma, leukocyte, and erythrocyte components. Plasma was collected following centrifugation of the heparinized blood at 2,000 × g for 5 min at 4 °C. The cell pellet was suspended to an original blood volume using HBSS and layered over a 34%/51% isotonic percoll discontinuous gradient and centrifuged at 800 × g for 20 min at 4 °C which was allowed to come to rest without the use of the centrifuge breaking system. Peripheral blood leukocytes were harvested from the 34%/51% interphase and erythrocytes from the pelleted material below the 51% layer. Cells were washed twice with HBSS, verified for >99% purity via hemocytometer, and suspended at a final concentration of 10e^7^ cells per 100 µL HBSS which was used for PRV qPCR screening.

### Passage of PRV via cohabitation or i.p. injection

PRV 16-005ND inoculate not used in the injection challenge above was thawed and administered to three sets of 15 fish (~150 g per fish) by intra-peritoneal injection, 200 µL per injection, under MS-222 anesthesia. Fish were cultured in 850 L tanks as above with the exception that the volume of 11 °C seawater was reduced to 250 L and supplied with a flow of 15 L per min. After a period of 1, 9, or 15 wpc, an equal number (n = 15) naïve cohabitants (with clipped adipose fins) were introduced to each to the three tanks. Blood samples were collected from six of both injected (shedder) and introduced (sentinel) fish after 4 and 8 weeks of cohabitation and screened for PRV L1 transcripts as described above.

### Comparative analyses

Trinity assembled PRV segments were concatenated and compared to two previously published PRV genomes (B5690 and V3621) by Jukes-Cantor phylogenetic relationship analysis using Geneious software. The relative quantities (scaled to the minimum value) of PRV L1 transcripts following differential pre-amplification denaturation were compared at 2, 4, and 10 wpc for both PRV 16-005ND and 16-011D challenged groups by one-way ANOVA and Tukey post-test of log-transformed data. The proportional change of PRV ssRNA (relative to total PRV RNA) was as assessed over time in both PRV 16-005ND and 16-011D challenged groups by one-way ANOVA and Dunnett’s multiple comparison post-test of arcsin-transformed values. The relative quantity (scaled to the minimum value) of each PRV 16-005ND RNA segment as well as the single-stranded mRNA proportion of each segment was assessed at 2, 4, and 10 wpc by two-way ANOVA with Bonferroni post-tests following log-(relative quantity) and arcsin-(proportional quantity) transformation. The contributing proportion of total PRV RNA and total single-stranded mRNA by each segment were considered independent and dependent to time, respectively, by two-way ANOVA. Thus, total PRV segment expression was pooled from 2, 4, and 10 wpc and compared by one-way ANOVA and Tukey’s post-hoc test of arcsin transformed values and single-stranded mRNA proportional expression was compared separately at each time point by one-way ANOVA with Dunnett’s multiple comparison post-test relative to L1. The trend in hematocrit of both PRV 16-005ND and 16-011D challenged fish were compared to controls by two-way ANOVA with Bonferroni post-tests of arcsine transformed values. This was similarly applied to comparing the quantity of erythrocyte cytoplasmic inclusion bodies observed in PRV 16-005ND versus 16-011D infected fish. All gene expression data was normalized to β-actin transcription. Normalized quantities were scaled to the minimum value for each gene prior to analysis of log-transformed data by two-way ANOVA with Bonferroni post-tests. Heart and skeletal muscle histopathology inflammation scores for PRV 16-005ND and 16-011D infected fish estimated at each time point throughout challenge was compared to control fish by Mann Whitney U tests without multiple comparison adjustment.

### Ethics statement

All work with animals was performed in strict accordance with the recommendations set out by the Canadian Council on Animal Care (CCAC) guide to the care and use of experimental animals and all live animal protocols were approved by the Pacific region animal care committee (animal use protocol number: 16–013). All fish handling was performed under Aquacalm™ (Syndel Laboratories Ltd.) or tricaine methanesulfonate (MS222) anesthesia.

## Supplementary information

Dataset 1

## Data Availability

Data presented in this manuscript are provided in Supplement 1, available through NCBI SRA SRP145317, or NCBI GenBank accessions MH347359 – MH347378.
