# Author Correction: Piscine orthoreovirus demonstrates high infectivity but low virulence in Atlantic salmon of Pacific Canada

**DOI:** 10.1038/s41598-020-61781-x

**Published:** 2020-03-10

**Authors:** Mark P. Polinski, Gary D. Marty, Heindrich N. Snyman, Kyle A. Garver

**Affiliations:** 1 Pacific Biological Station, Fisheries and Oceans Canada, Nanaimo, V9T 6N7 Canada; 2 Animal Health Centre, Ministry of Agriculture, Abbotsford, V3G 2M3 Canada

Correction to: *Scientific Reports* 10.1038/s41598-019-40025-7, published online 13 March 2019

In this Article, the authors neglected to include some professional collaborations that may be perceived as potential conflicts of interest. The Competing Interests section should read:

“In their capacity as Government of Canada research scientists, MP.P. and K.A.G. have had recent or ongoing collaborations with the following institutions that have the potential to be financially affected by the findings of this study: British Columbia Centre for Aquatic Health Sciences, British Columbia Salmon Farmers Association, Canadian Food Inspection Agency, Centre for Aquaculture Technologies Canada, Centre for Coastal Health, Cermaq Canada, Freshwater Fisheries Society of British Columbia, Grieg Seafood, Mowi Canada West, New Jersey Division of Fish and Wildlife, Northwest Indian Fisheries Commission, Norwegian Veterinary Institute, Okanagan Nation Alliance, Pacific Salmon Commission, United States Geological Survey, and the Washington Department of Fish and Wildlife. These institutions did not provide financial support for this study, participate in its experimental design, contribute to data collection, analysis, interpretation, preparation of the manuscript, or decision to publish. All authors declare they have no commercial interests with relevance to this work.”

